# Functional characterization of the tomato *HAIRPLUS* gene reveals the implication of the epigenome in the control of glandular trichome formation

**DOI:** 10.1093/hr/uhab015

**Published:** 2022-01-28

**Authors:** Rocío Fonseca, Carmen Capel, Fernando J Yuste-Lisbona, Jorge L Quispe, Cristina Gómez-Martín, Ricardo Lebrón, Michael Hackenberg, José L Oliver, Trinidad Angosto, Rafael Lozano, Juan Capel

**Affiliations:** 1 Centro de Investigación en Agrosistemas Intensivos Mediterráneos y Biotecnología Agroalimentaria (CIAIMBITAL), Universidad de Almería, Carretera de Sacramento s/n, 04120 Almería, Spain; 2 Department of Genetics, Faculty of Science, University of Granada, Campus de Fuentenueva s/n, 18071 Granada, Spain; 3 Laboratory of Bioinformatics, Centro de Investigación Biomédica, PTS, Avda. del Conocimiento s/n,18100 Granada, Spain

## Abstract

Trichomes are specialised epidermal cells developed in the aerial surface of almost every terrestrial plant. These structures form physical barriers, which combined with their capability of synthesis of complex molecules, prevent plagues from spreading and confer trichomes a key role in the defence against herbivores. In this work, the tomato gene *HAIRPLUS* (*HAP*) that controls glandular trichome density in tomato plants was characterised. HAP belongs to a group of proteins involved in histone tail modifications although some also bind methylated DNA. *HAP* loss of function promotes epigenomic modifications in the tomato genome reflected in numerous differentially methylated cytosines and causes transcriptomic changes in *hap* mutant plants. Taken together, these findings demonstrate that HAP links epigenome remodelling with multicellular glandular trichome development and reveal that *HAP* is a valuable genomic tool for pest resistance in tomato breeding.

## Introduction

Plants exhibit a large number of adaptive traits that allow them to succeed in their environments. Among these, the formation of epidermal protrusions called trichomes is one of the most recurrent. Trichomes are epidermal structures which can be classified as uni- or multi-cellular based on their cell number, and as glandular or non-glandular depending on their secretory capability [1]. Trichomes are involved in abiotic stress responses, including extreme temperatures or excessive radiation, and mainly in biotic stress response including pest resistance [2, 3].

Trichomes of the model species *Arabidopsis thaliana* are unicellular and non-glandular [4], and the genetic network controlling their initiation is well known. One of this network’s key regulator is *GLABRA1* (*GL1*), which encodes a R2R3 MYB transcription factor whose loss of function mutation results in a complete absence of trichomes [5]. The MYB-domain of GL1 physically interacts with the N-terminal domain of the proteins encoded by *GLABRA3* (*GL3*) and *ENHANCER OF GLABRA3* (*EGL3*), two bHLH proteins that promote trichome formation in a redundant manner [6, 7]. In addition, *TRANSPARENT TESTA GLABRA 1* encodes a protein with four conserved WD-domains which interacts with both GL3 and EGL3, and together with GL1, forms a multimeric complex that triggers trichome initiation in *A. thaliana* [7]. The *GLABRA2* (*GL2*) gene appears to be the downstream target of this multimeric complex, since its promoter contains a MYB binding site to which GL1, as part of the molecular complex, can bind to activate *GL2* transcription [6]. On the other hand, four MYB proteins encoded by *CAPRICE* (*CPC*), *TRIPTHYCHON* (*TRY*), *ENHACER OF TRY AND CPC1* (*ETC1*) and *ENHACER OF TRY AND CPC2* (*ETC2*), repress trichome initiation in a redundant manner by interacting with GL3 [8]. As a result of this interaction, GL1 cannot bind to GL3 and the activation complex is not formed [6].

Whether there is a similar multimeric complex controlling trichome formation in Asterid species, and particularly within the Solanaceae family, remains unclear. However, the *Mixta-like* genes, which code for MYB-related transcription factors, seem to hold a conserved role in trichome initiation. The *Mixta* gene was identified in *Antirrhinum majus* and is well known for inducing ectopic trichome formation on the anthers of the woody nightshade *Solanum dulcamara* [9], as well as for promoting multicellular trichome and conical cells formation in *Nicotiana tabacum* [10]*.* New roles have been described for a *Mixta* homologous gene in epidermal trichome patterning of tomato plants. Thus, silencing of *SlMixta-like* induces the formation of trichome clusters in the leaf surface whereas leaves of knock-out lines of this gene exhibit a higher trichome density [11]. Given that neither the ectopic expression of *Mixta* in *A. thaliana* [12], nor overexpression of *GL1* in tobacco [10] have any effect on trichome formation, it has been suggested that trichome initiation in *A. thaliana* and Solanaceae species might follow divergent regulatory pathways [13].

Five types of trichomes have been described for cultivated tomato (*Solanum lycopersicum* L.) [14]: type-I is 2–3 mm long, has a multicellular base and a glandular small cell in the tip; type-III is non-glandular and 0.4–1 mm long with a unicellular flat base; type-V also has a unicellular and flat base and is 0.2–0.4 mm long; type-VI are short trichomes with two stalk cells and a glandular head composed of four secretory cells and type-VII are very small trichomes (0.05 mm) with glandular heads formed by 4 to 8 cells (Supplementary Fig. S1). Wild tomato species develop type-II and -IV trichomes, the latter being characterised by a single cell flat base and a short stalk that connects with a glandular head (Supplementary Fig. S1). The presence of type-IV trichomes has been described as a heterochronic trait in the cultivated species, since they can only be detected at early developmental stages [15]. Non-glandular trichomes act as a mechanical barrier that prevents pest movement along the plant whereas glandular ones produce specialised metabolites that are toxic or deterrent to the pest or entrap them [16].

Despite their importance in pest resistance, very few trichome mutants that allow to shed light on the genetic regulation of multicellular trichome initiation have been described in tomato. One of those mutants is *hairless*, characterised by a distorted morphology of type-I trichomes and a deficient accumulation of sesquiterpenes in type-VI trichomes [17]. *HAIRLESS* codes for SRA1, one of the subunits in the SCAR/WAVE multiprotein complex that controls actin filament nucleation and polymerization [17]. Recently, the *hairless-2* mutant has been characterized and mutation in the *NAP1* gene, another component of the WAVE complex, proved to account for this mutant phenotype [18]. Furthermore, physical interaction among Hl1 and Hl-2 was detected as well as implication of SlHDZIV8, an HD-Zip IV transcription factor, in the regulation of *Hl-2* expression. All these findings reinforce the role of the SCAR/WAVE complex in the regulation of tomato multicellular trichome formation [18].

Another tomato mutant, *hair absent*, which exhibits a complete absence of type-I trichomes on the epidermis, has also been characterised, and encodes a C2H2 zinc-finger protein [19]. On the other hand, the *woolly* (*wo*) mutant is characterised by an increase in type-I trichome density and embryo lethality [20, 21]. *Wo* encodes an HD-Zip protein containing a START-domain that physically interacts with a B-type cyclin named SlCycB2 necessary for the development of multicellular trichomes [22]. Transcriptomic analysis performed in *wo* and non-*wo* plants has allowed to identify a wide number of lncRNAs differentially expressed in both genotypes [23]. Among them, lncRNA000170 was found to be highly expressed in stem trichomes of *wo* plants and transgenic experiments confirmed that overexpression of this lncRNA inhibits type I trichome formation and downregulated key trichome regulators as *Wo*, *Hair*, *SlCycB2*, and *SlCycB3* [23]. All these findings demonstrate the existence of genetic networks and protein complexes in tomato of a very different nature from genetic network and the protein complex which regulates trichome initiation in *Arabidopsis*.

In this work, a tomato mutant that exhibits a high type-I trichome density, which has been named *hairplus* (*hap*), is described and characterised. Fine mapping and genetic complementation analysis showed that *HAP* encodes a SUVH3 histone lysine methyltransferase. This protein family is mainly composed of histone tail modifying proteins, although some bind methylated DNA, all playing key roles in epigenetic control of gene expression [24, 25]. The present findings identify a novel tomato regulator of trichome formation and shed light on the role of SUVH3 histone methyltransferases in transcriptional control mediated by epigenomic modifications.

## Results

### *Hairplus* mutation overall impairs trichome density

**Figure 1:**
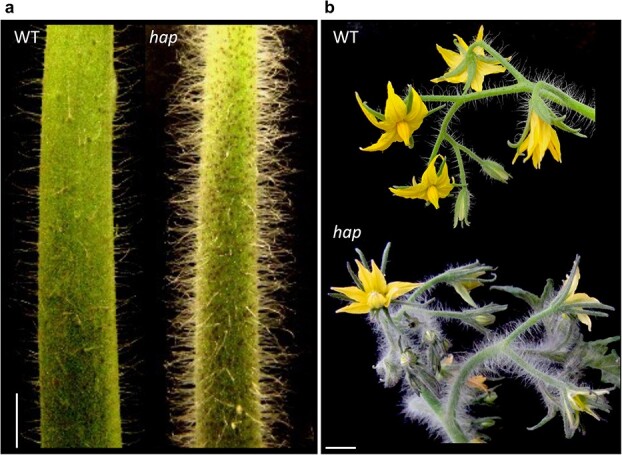
The *hap* mutant phenotype. (a) Main vegetative stem of a wild-type (WT) Moneymaker plant and a *hap* mutant plant with increased trichome density. (b) Inflorescence stem of a WT plant and a *hap* mutant plant where higher trichome density can be appreciated. Scale bars apply to 1 cm.

The *hap* mutant was characterised as part of a mutant collection obtained in tomato (*S. lycopersicum* L., cv. Moneymaker) using ethylmethanesulfonate (EMS) as a mutagen agent. This mutant’s most conspicuous phenotype is a higher trichome density in vegetative stems and inflorescence stems when compared to wild-type (WT) plants (Fig. 1).

Additionally, mutant plants develop plant shoots on the older leaf petioles and exhibit a lower growth than WT plants (Supplementary Fig. S2), without any other morphological change in vegetative, i.e. roots, or reproductive organs, i.e. flowers and fruits. The segregation ratio observed in a M_2_ segregating population was consistent with a monogenic recessive inheritance for the *hap* mutant phenotype (202 WT: 76 mut; *χ*^2^ = 0.81, *p* = 0.36).

**Figure 2:**
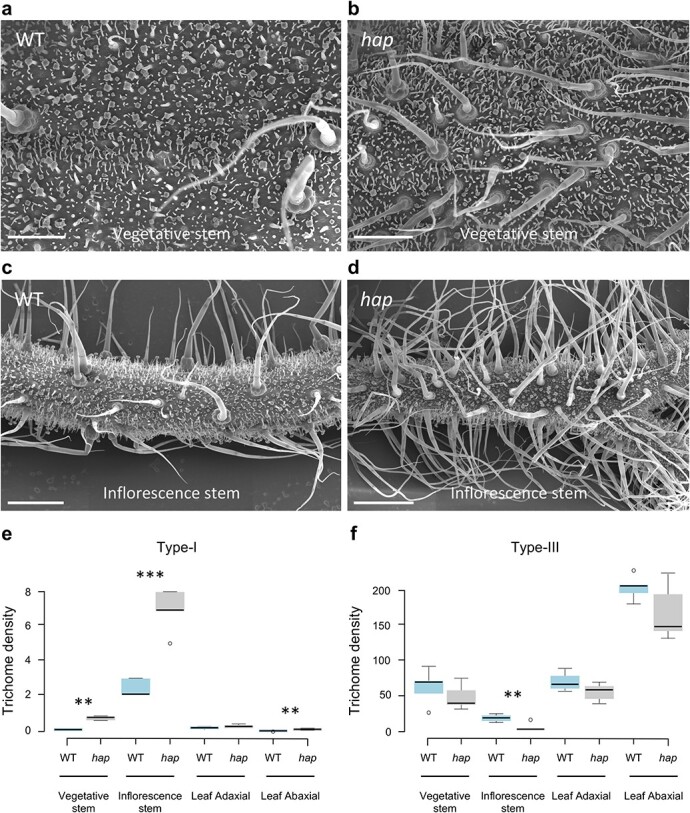
Trichome density in WT and *hap* mutant plants. (a) Vegetative stem of WT plants. (b) Vegetative stem of a *hap* mutant plant where higher trichome density can be observed. (c) Inflorescence stem of a WT plant. (d) Inflorescence stem of a *hap* mutant plant showing higher trichome density. (e) Type-I trichome density in vegetative stems, inflorescence stems and leaf adaxial and abaxial side of WT and *hap* mutant plants. (f) Type-III trichome density in vegetative stems, inflorescence stems and leaf adaxial and abaxial side of WT and *hap* mutant plants. In (e) and (f) centre lines show the average values of trichome density (trichomes / mm^2^), whereas box limits indicate the 25th and 75th percentiles as determined by R software. Whiskers extend 1.5 times the interquartile range from the 25th and 75th percentiles and outliers are represented by dots. Two-ways ANOVA was performed in order to assess genotype and tissue effect on trichome density (P < 0.01). Statistical differences among WT and *hap* plants were assessed by means of a post-hoc Least Significant Difference test (LSD). ** Significant differences at P < 0.01. *** Significant differences at P < 0.001. Scale bars apply to 0.5 mm (a, b) and to 1 mm (c, d).

In order to determine the trichome types characterising the *hap* phenotype, trichome identity and density of WT and *hap* mutant plants were examined using scanning electron microscopy (SEM). Mutant plants exhibit a higher density of long, multicellular base, type-I trichomes than WT plants, and significant differences were observed in the main vegetative stem (Fig. 2a, 2b, 2e), inflorescence stems (Fig. 2c, 2d, 2e) as well as in the abaxial side of leaves (Fig. 2e).

In addition, significant differences were also observed as regards to type-III trichome density, although in an opposite way, since density decreased in the inflorescence stems of *hap* mutant plants when compared to WT ones (Fig. 2f).

Finally, *hap* mutant plants do not differ from WT in the density of the very abundant multiglandular type-VI trichomes, either on other features related to trichome development (i.e. type-V and type-VII trichomes), including stomata density (Supplementary Fig. S3), or size, number or identity of the epidermal cells. Additionally, aberrant branched type-I trichomes were observed in *hap* mutant at different maturation stages (Supplementary Fig. S4). These results suggest that *HAP* controls trichome formation and particularly type-I trichomes in vegetative and reproductive organs.

### Mutant plants show decreased pest damage

**Figure 3:**
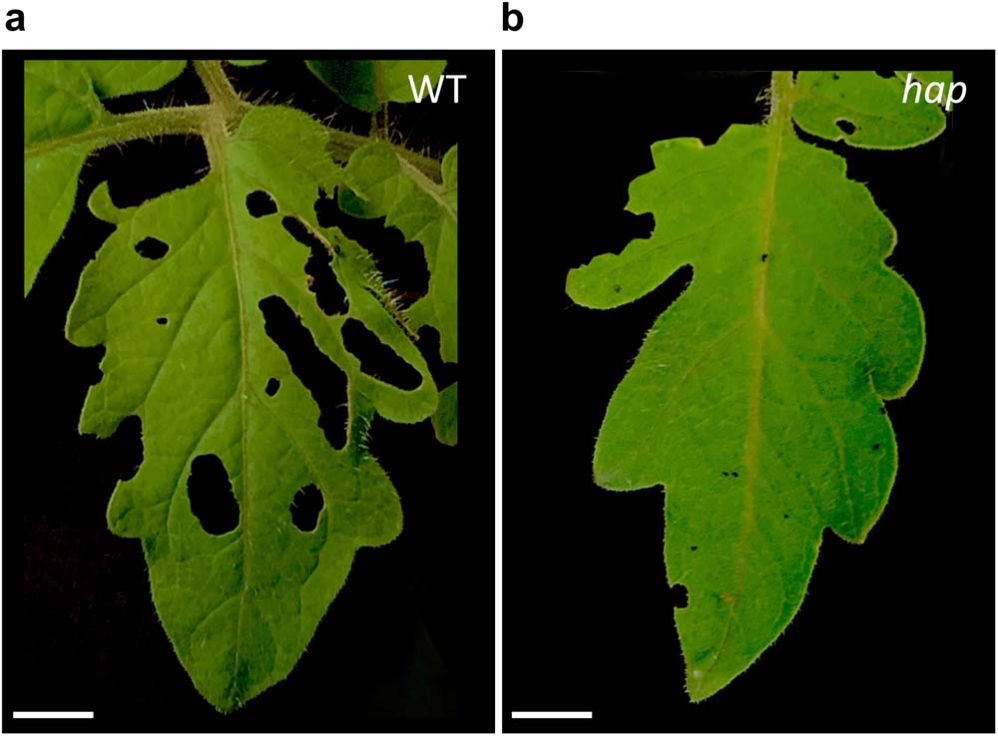
Damage caused by the tomato fruit borer Helicoverpa armigera (Hübner) after 15 days feeding in WT and *hairplus* mutant plants. (a) WT plants show severe damage by the larvae feeding, which causes large lesions in the leaf. (b) hap mutant plants, on the other hand, exhibit higher resistance levels, since larvae feed only in the leaf margin.

With the aim to test if the increased density of type I glandular trichomes observed in mutant plants was related to an increase in pest resistance, we used larvae of the lepidopteran *Helicoverpa armigera* (Hübner) in a no-choice fed trial. Our results showed that while in control MM plants larvae fed in the entire leaf surface, in mutant plants damage was mainly restricted to the leaf margin (Fig. 3a, b).

At the end of the pest resistance trial, leaf damage percentage in WT plants was 59.2 ± 12.6% whereas in mutant plants was significantly lower (17.5 ± 3.53). This result indicates that *hap* increased density of type I glandular trichomes may be related to an increment of tomato pest resistance.

### *HAP* encodes a SET-domain SUVH putative histone lysine methyltransferase

**Figure 4:**
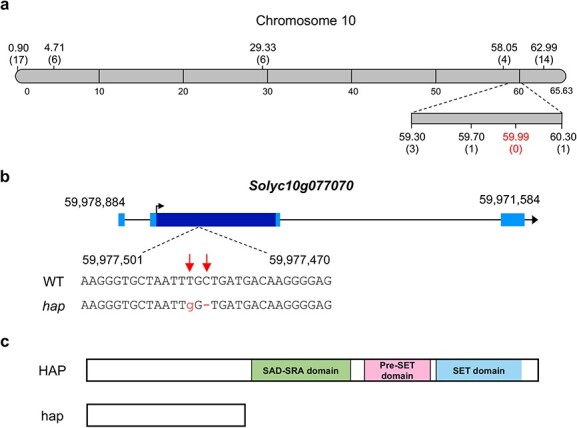
Map based cloning of *HAP*. (a) Fine mapping delimited *HAP* to an interval of 600 Kb of chromosome 10. Numbers in parenthesis indicate the number of recombinant chromosomes identified between the *HAP* gene and each genetic marker analysed. (b) Sequencing data obtained from a pool of DNA from *hap* mutant plants allowed for the identification of a deletion followed by a point mutation in *Solyc10g077070*, a gene that encodes for a putative histone lysine methyltransferase. The arrow indicates the translation start site. Coding sequence is darker than non-coding sequences. (c) Schematic representation of the HAP protein domains. Mutations in *hap* give rise to a premature stop codon and, as a result, the putative hap mutant protein lacks the functional domains characteristic of this gene family.

In order to isolate the *HAP* gene, a mapping population was generated by crossing a *hap* mutant plant with a plant from the wild relative species *Solanum pimpinellifolium* accession LA1589, used as pollen donor due to its small density of type I trichomes. F_1_ was self-pollinated and the F_2_ population was phenotypically characterised. Linkage mapping was performed using codominant markers distributed along the genome [26], which allowed for the location of the *hap* mutation to an approximately 0.6 Mb fragment on chromosome 10 (Fig. 4a).

The precise chromosome location of the mutation was defined through mapping by sequencing; with this aim, two DNA pools, each consisting of equimolar amounts of DNA from 14 F_2_ homozygous plants of WT and mutant phenotypes respectively, were sequenced. Analysis of the segregating allele frequencies confirmed the location of the mutation on chromosome 10 (Supplementary Fig. S5) and allowed to identify in the mapping interval two mutations in the coding sequence of *Solyc10g077070* gene, where a deletion followed by a single-nucleotide variant causes a premature stop codon (Fig. 4b). This gene codes for a putative histone 3 lysine-9 methyltransferase homologue of the SET (Suppressor of variegation, Enhancer of zeste and Trithorax) [27] -domain containing SUVH (suppressor of variegation 3–9 homologue) [28] proteins of *A. thaliana*, and as a result of these mutations, the putative HAP mutant protein lacks all functional domains characterising this group of methyltransferases, including the SAD-SRA (SET-associated *Deinococcus* endonuclease domain, SET and Ring finger Associated) [28] and the SET-domain, which in turn are crucial for SUVH proteins’ activity [24] (Fig. 4c).

**Figure 5:**
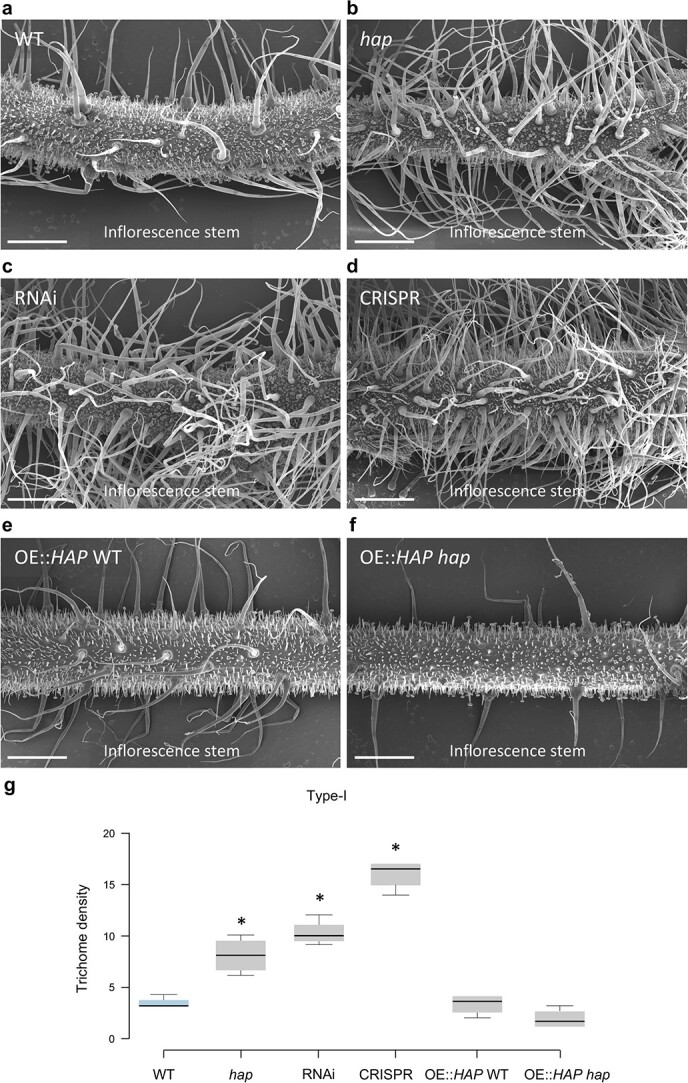
Modulation of type-I trichome density by *HAP* gene expression. (a) Inflorescence stem of a WT plant. (b) Inflorescence stem of a *hap* mutant plant. (c) Phenotype of a *HAP* RNAi silencing line showing increased type-I trichome density. (d) Phenotype of the inflorescence stem of a CRISPR-Cas9 knock-out line where the increase in type-I trichome density is even more evident. (e) Inflorescence stem of an overexpression line obtained in a WT background shows no difference when compared to WT. (f) Inflorescence stem of an overexpression line obtained in a *hap* mutant background showing less type-I trichome density than the observed in WT plants. (g) Type-I trichome density (trichomes / mm^2^) accounts, with lines showing the average values whereas box limits indicate the 25th and 75th percentiles as determined by R software. Whiskers extend 1.5 times the interquartile range from the 25th and 75th percentiles and outliers are represented by dots. Overall genotype effect was assessed by means of a One-way ANOVA test (P = 2.00•10–16), whereas significant differences were accounted using a Dunnett’s test for a significance value of P < 0.01 (*). All scale bars apply to 1 mm.

To confirm that the mutations in *Solyc10g077070* are responsible for the *hap* mutant phenotype, gene silencing experiments were carried out by means of an RNA interference (RNAi) strategy. Eleven independent diploid transformants were obtained and used for phenotypic characterisation. Expression analysis performed by quantitative RT-PCR showed that *Solyc10g077070* expression levels were drastically reduced in all RNAi T_0_ lines, ranging from 0.04 to 0.27 relative expression of that observed in WT plants (Supplementary Fig. S6). All T_0_ RNAi lines showed increased trichome density in inflorescence stems (Fig. 5c) and all have a similar phenotype to that of the *hap* mutant (Fig. 5b) suggesting that knocking-down of the gene is enough to produce the mutant phenotype.

Knockout mutations of *Solyc10g077070* were also engineered using the CRISPR-Cas9 genome editing system, and the four diploid T_0_ lines obtained were characterised by sequencing the region where the sgRNA was designed (Supplementary Fig. S7). The four T_0_ lines were biallelic mutants, and their phenotypes either resembled the *hap* mutant phenotype or even showed a more extreme phenotype, i.e. a greater type-I trichome density than the original *hap* mutant plants (Fig. 5d). Finally, overexpression of the *Solyc10g077070* gene produced no phenotypic changes in WT Moneymaker plants (Fig. 5e) but restored the WT phenotype in *hap* mutant plants (Fig. 5f), demonstrating that a WT copy of the gene complemented the mutant phenotype, and suggesting that the simultaneous presence of a truncated form of HAP with an excess of the protein’s WT copies changes HAP functionality. Accounts of type-I trichome density in these transgenic lines confirmed the significant increase observed in RNAi and CRISPR-Cas9 knock out lines when compared to WT and even with *hap* mutant plants (Fig. 5g).

Furthermore, T_1_ advanced RNAi progenies also resembled the *hap* mutant phenotype, whereas T_1_ CRISPR plants azygous for the presence of the CRISPR-Cas9 construct but homozygous mutant for the edited alleles shows higher trichome density than the *hap* mutant (Supplementary Fig. S8). Taken together, these results support that *Solyc10g077070* is responsible for the *hap* mutant phenotype, and therefore, hereinafter it will be referred to as *HAP*.

### Gene expression changes promoted by *hap* mutation

With the aim to determine the expression pattern of *HAP*, quantitative RT-PCR was performed using total RNA extracted from several tissues of WT plants, which included root, stem, inflorescence stem, flower buds, whorls of anthesis day flowers and fruits at different maturation stages. Expression patterns confirmed that *HAP* is constitutively expressed in all analysed tissues, reaching the highest expression level in adult leaf followed by the inflorescence stem. The lower levels of the gene’s transcripts were observed in sepals of anthesis day flowers and fruits in breaker state (Supplementary Fig. S9a).

The expression of the genes previously reported as involved in tomato trichome development, i.e. *Hair* [19], *Woolly* [21], *SlCycB2* [22], *Hairless* [17], *SlMixta-like* [11] and *SlMYC1* [29] was analysed in inflorescence stems of WT and *hap* mutant plants with the purpose to explore possible genetic interactions of *HAP*. The results obtained showed a slight decrease in the level of transcript of *Hairless* and *SlMYC1* in *hap* mutant. However, no significant differences in relative expression were found among WT and *hap* mutant plants regarding the analysed genes, except for *HAP* itself*,* which was significantly repressed in *hap* mutant plants (Supplementary Fig. S9b). Downregulation of *Hairless* or *SlMYC* does not alter type I trichome density but instead causes the formation of malformed trichomes [17] or decreases type VI trichomes density [29] respectively. These results suggest that *HAP* controls type-I trichome density by a signal transmission pathway in which changes in the transcription of the analysed genes seem not to be involved.

Thus, the molecular signalling cascade downstream of *HAP* was investigated using RNA sequencing of *hap* and WT inflorescence stems. This analysis allowed to identify 82 downregulated and 8 upregulated genes in mutant plants that showed at least a twofold change in transcript number (Supplementary Tables S1 and S2). Among the upregulated genes, was found *Solyc12g096570* which is a homologue of the *Arabidopsis ARGOS*, a gene highly induced by auxins that plays a key role in aerial organs size control [30]. Another upregulated gene in *hap* is *Solyc02g085910*, which codes for a homologue of the *Arabidopsis* LOB-domain containing protein; when ectopically expressed, it leads to alterations in the size and shape of leaves and floral organs as wells as to male and female sterility [31]. One of the barely expressed genes in WT plants but induced in *hap* mutant plants is *Solyc03g082550*, which codes for a Homeobox leucine zipper protein homologue of an *Arabidopsis* protein family implicated in the ABA response network and cell growth control [32, 33].

Four transcription factors were downregulated in *hap*, *Uniform ripening* (*Solyc10g008160*), that codes for a Golden 2-like transcription factor regulating tomato fruit chloroplast development [34], two Ethylene Responsive transcription factors (*Solyc01g090300* and *Solyc11g011740*), and a Dof zinc finger transcription factor coded by *Solyc08g008500*. In addition, five genes (*Solyc09g092490*, *Solyc10g085870*, *Solyc10g085880*, *Solyc12g088700*, *Solyc12g098600*) of the multigene gene family that code for UDP-glucosyltransferase belonging to the family 1 of Glycosyltransferases protein [35], were also found repressed in the *hap* mutant. On the other hand, the *HAP* gene is also downregulated in the *hap* mutant transcriptomic analysis which supports the results obtained by quantitative expression analyses.

### *HAP* induces epigenetic changes in the tomato genome

**Figure 6:**
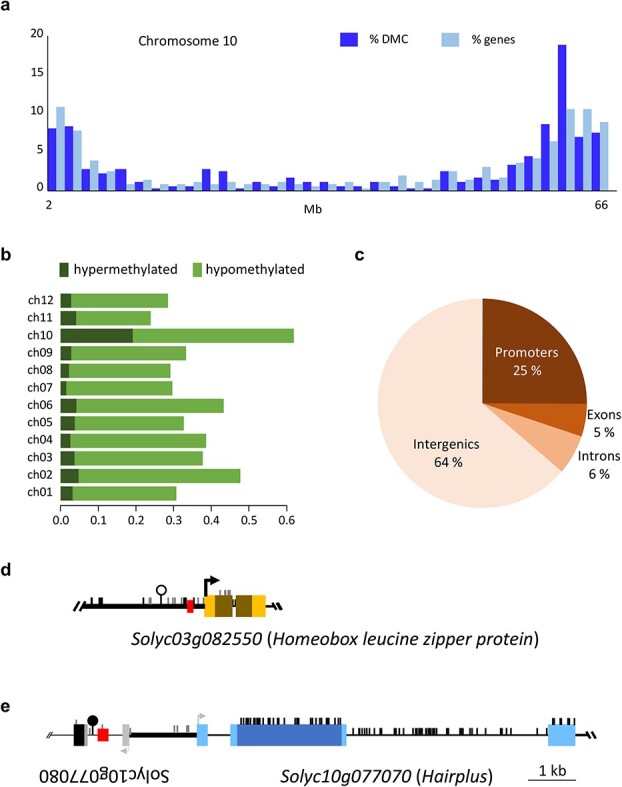
Location of DMCs in *hap* mutant plants. (a) Density of DMC and genes over chromosome 10. Each bar represents the percentage of DMCs or genes in 2 Mb. (b) Percentage of hyper (dark green) or hypo- (light green) methylated DMCs on the 12 *hap* chromosomes. (c) Annotation of the *hap* DMCs. (d) Genomic organization of the *Solyc03g082550* gene and (e) of the *HAP* gene. d and e, boxes represent exons and thin horizontal lines represent introns; intergenic sequences are depicted as tick black lines; annotated LTRs are represented as red boxes; methylated CpG are represented as vertical lines. Hypomethylated DMC is represented as a white circle whereas hypermethylated CpG is represented as a black circle. The arrows indicate the transcription start sites. Coding sequence are darker than non-coding sequences.

*Arabidopsis* proteins homologue to HAP seem to have no histone methyltransferase activity yet bind methylated sequences near transposons thus promoting demethylation and transcriptional activation of genes located near such transposable elements [36]. No histone methyltransferase activity of HAP (Supplementary Fig. S10) was found, so we decided to analyse the epigenetic modifications caused by *HAP’s* loss of function through the whole-genome bisulfite sequencing of genomic DNA from the same inflorescence stems of *hap* and WT plants analysed by RNA-seq. As a result, differentially methylated cytosines (DMCs) between WT and mutant phenotypes were found by means of the logistic regression function implemented in *methylKit* [37] through comparison of the sequences from the 3 replicates of WT to the 3 replicates of *hap* mutant DNA. A total amount of 2113 DMCs was obtained (*q*-value <0.01 and percent methylation difference larger than 25%), from which 270 were hyper- and 1843 hypo-methylated cytosines in *hap* mutant plants. The DMCs identified in the genome of *hap* mutants are not randomly located as indicated by the significant correlation with the well-known non-random distribution of genes, being DMCs mainly located in the euchromatic telomeric portion of the 12 tomato chromosomes (Fig. 6a and Supplementary Fig. S11).

Chromosome 10 contains the highest amount of hypo- and hypermethylated DMCs, whereas chromosome 11 contained the smallest number of DMCs. The rest of chromosomes contained a similar number of DMCs (Fig. 6b). Although DMCs are mainly located in gene-rich portions of the genome, most of them (64%) are located in intergenic regions, 25% located in promoters and 11% in transcribed regions from which 5% were found in exons and 6% in introns (Fig. 6c and Supplementary Fig. S12). There is no statistically significant correlation between the position of the DMCs and the DEGs, probably due to the fact that most of the DMCs were found far away from identified DEGs, except for *Solyc03g082550*, upregulated in *hap*, which codes for a Homeobox leucine zipper protein and holds a DMC in its promoter which is demethylated in *hap* and hypermethylated in WT plants (Fig. 6d). Among the down-regulated genes found in *hap* mutant plants, 5 include at least one DMC in their transcribed sequences (*Solyc01g068460*, *Solyc03g097870*, *Solyc04g007400*, *Solyc07g006680*, *Solyc11g071290* and *Solyc12g098600*) and 3 genes (*Solyc04g052980*, *Solyc06g052010* and *Solyc10g077070*-*HAP*) have at least one DMC in their promoters. *HAP* gene itself (*Solyc10g077070*) has a DMC 2 Kb 5′ upstream, in a region that can be considered as its promoter. This DMC is located near an LTR and was found hypermethylated in the *hap* mutant (Fig. 6e) suggesting a possible role of this epigenetic mark in the regulation of the *HAP* gene.

### HAP homologues are conserved through evolution but have diverged with novel functions

As mentioned above, tomato HAP is homologous to a group of SET-domain containing SUVH proteins linked to epigenetic control of gene expression [36, 38]. In the model species *A. thaliana*, 29 genes coding for SET-domain containing proteins have been identified [39]. With the aim to characterise the phylogenetic relationship among these proteins and the tomato SUVH proteins including HAP, a Multiple Sequence Comparison was first computed and then a neighbour joining tree was constructed. The most closely related proteins to HAP, the tomato protein coded by the *Solyc09g082050* gene located in chromosome 9 and four *Arabidopsis* SUVH proteins, were grouped into the same clade (Supplementary Fig. S13). Notably, multiple alignment showed that these proteins share a small region of 8 amino acids within the SET-domain (GWGLRSWD) that is exclusive to this clade (Supplementary Fig. S14). None of the four *Arabidopsis* genes homologous to *HAP* showed phenotypic alterations when they were mutated [36, 40]. However, the lack of function of the SET-domain protein HAP changes trichome density suggesting a divergent role respect to its *Arabidopsis* homologues.

## Discussion

Even though trichomes are epidermal structures common in plants, genetic networks controlling their development appear to differ among species [6]. Several studies have been carried out over the last decades in the model species *A. thaliana*, leading to the characterisation of a molecular complex of transcription factors that controls trichome development [8]. In this complex, a WD40 transcription factor (TTG1) interacts with bHLH transcription factors (GL3, EGL3), while a R2R3-MYB transcription factor (GL1) competes with single-repeat MYBs (CPC, TRY, ETC) for binding to the bHLH proteins to initiate the formation of a trichome [6]. It is worthy of mention that these protein complexes seem to be exclusive of *Arabidopsis* since protein complexes formed by proteins homologous to these have not been found in other species so far. Nevertheless, very little information exists regarding the genetic network regulating trichome development in tomato and other Solanaceae species. *Hair* and *Woolly* are among the few genes already characterised in tomato both encoding transcription factors required for type-I trichome differentiation. Physical interaction among both transcription factors, the single C2H2 zinc-finger HAIR and the Homeodomain WOOLLY, has been proven and is evidence for the existence of novel protein interactions controlling trichome development in tomato [19]. WOOLLY physically interacts as well with the SlCycB2 cyclin to control type-I trichome density [13]. These results suggest that a protein complex HAIR-WOOLLY-SlCycB2 may exist in tomato but has not been detected yet. Another gene recently demonstrated to act as a negative regulator of trichome initiation is *SlMixta-like*, which codes for a R2R3-MYB transcription factor [11], corroborating that the protein complex controlling tomato trichome development must be similar to but different from those described in *Arabidopsis*. In this work, *HAP* has been characterised as a novel gene involved in type-I trichome formation most likely through a novel tomato genetic network, since none of the regulatory genes previously described showed significant alterations in their transcript levels in *hap* mutant plants. The RNA sequencing experiment performed in *hap* mutant plants identified the deregulation of tomato genes never related to the formation of trichomes in the past. Furthermore, branched type-I trichomes have been observed in the surface of older *hap* mutant leaves in a similar way to that of ramose type-I trichomes described in the *woolly* mutant [15]. *Wo* has been described to promote multicellular type-I trichome formation by up-regulating *SlCycB2* expression, which in turn induces a shift from endoreduplication to mitosis [13]. Thus, *HAP* might act in a similar way to *Wo* by promoting changes in the cell cycle that can ultimately induce mitotic divisions. However, *hap* mutant plants do not show significant changes in the transcript levels of either *Wo* or *SlCycB2* genes, suggesting the existence of different genetic networks controlling the branching of type-I trichomes.

In *A. thaliana*, the *KRYPTONITE* (*KYP*) gene codes for SUVH4, a methyltransferase specific for Lysine 9 of histone 3 (H3, Lys9), which acts as a suppressor of the *SUPERMAN* (*SUP*) locus [41]. Mutant plants of the *sup* alleles *clk-st* showed defects in the number of floral organs due to the extreme methylation of *SUP*. The *kyp* loss-of-function mutants did not exhibit morphological alterations, although a reduction in *SUP* methylation level is observed in CpG and especially CpHpG sites. Thus, it could be inferred that SUVH methyltransferases promote gene silencing. The closest *HAP* homologue gene in *Arabidopsis* (*At1g73100*) codes for SUVH3, which has a partially redundant function with its homologue SUVH1 (*At5g04940*) [36, 38]. None of the *Arabidopsis* homologues SUVH1 and SUVH3 show *in vitro* histone methyltransferase activity [36], which is similar to the observed for HAP as it has been assessed in our results (Supplementary Fig. S10). However, it has been proven that SUVH1 and SUVH3 interact with the DNAJ-domain containing proteins forming a protein complex that binds to methylated sites near transposable elements (TEs), inducing the demethylation and transcriptional activation of the genes proximal to these TEs [36, 38, 42]. Moreover, *suvh1* and *suvh3* mutants as well as double mutants do not show morphological alterations and their overall genomic methylation levels also appear unaltered [36, 38]. Nevertheless, *HAP* loss of function induces a large number of epigenetic changes in the tomato genome. Since the DMCs identified in *hap* mutant plants are located in all the chromosomes, and given the large number of down-regulated genes (82) in these plants, HAP might form a protein complex inducing the transcription of the promoter methylated genes instead of functioning as a histone methyltransferase. Further studies will focus on dissecting whether HAP is capable of recognizing RNA-dependent methylated regions and of recruiting proteins to these sites of the genome. A candidate genomic region where HAP could modulate CpG methylation is its own promoter, where the *hap* genome contains a DMC hypermethylated located in the proximity of an LTR (Fig. 6e). This latter DMC, together with the DMC located in the promoter of the Homeobox coding gene *Solyc03g082550*, could be considered as CpG traffic lights, epigenetic marks correlated to the expression level of the closest gene [43]. Further characterisation of the HAP genomic targets as well as the identification of its protein interactors may identify new components of the tomato protein complex controlling glandular trichome formation that will be used in future tomato breeding programmes.

Trichomes are protuberances of epithelial cells with multiple functions, with pest defence being one of the most proven. Thus, an increase in glandular trichome density has been correlated with resistance to plagues in the cultivated and wild tomato species. Such is the case of type-IV glandular trichomes, which have been reported to be part of the resistance mechanisms to lepidoptera and spider mite pests, since they are related to an increase in entrapment of some plagues [44] or to resistance to spider mite *Tetranychus urticae* (Koch) [45, 46]. In the tomato wild-relative *Solanum galapagense*, a negative correlation has been observed between the presence of glandular trichomes of types I, IV and VI and adult survival, oviposition rate and pre-adult survival of the whitefly *Bemisia tabaci* [47]. Given that the *hap* mutant exhibits a higher density of glandular type-I trichomes in leaves and vegetative as well as reproductive stems, an increase in pest resistance was expected. Data obtained while assessing *hap* mutant resistance to the tomato fruit borer *Helicoverpa armigera* (Hübner) indicate that feeding damage is reduced in mutant plants when compared to WT ones (Fig. 3). Future works should focus on demonstrating that the reduced feeding damage is correlated with pest resistance of *hap* mutant plants, and whether it is related to a higher amount of exudates or to the physical barriers that the trichomes represent in mutant plants.

## Materials and methods

### Plant material and growth conditions

The *hairplus* mutant was identified as part of a mutant collection obtained by chemical mutagenesis with ethylmethanesulfonate (EMS) in *S. lycopersicum* cultivar Moneymaker. Seeds were incubated in a solution containing 0.7% of EMS (Sigma-Aldrich) at 30°C with gentle shaking for 16 hours. Afterwards, seeds were thoroughly washed in distilled water and sown in seedling beds. Germination was assessed prior to transplant. M_2_ seeds were obtained by self-pollination of M_1_ plants under greenhouse growth conditions. Wild relative *S. pimpinellifolium* accession LA1589 obtained from Tomato Genetics Resource Center (http://tgrc.ucdavis.edu/), was used for generating F_2_ mapping population. Phenotypic characterisation of *hap* mutant was carried out in M_2_ populations grown along with control WT plants. All experiments were conducted under greenhouse conditions and following standard management practices, including regular fertilization.

### Scanning electron microscopy

Trichome density and morphology was assessed in vegetative stems, inflorescence stems and leaves of the *hairplus* mutant and WT plants. Five mutant and five control samples of all these tissues were analysed by Scanning Electron Microscopy conducted as previously described [48]. Briefly, samples were fixed in FAEG (3.7% formaldehyde, 5.0% acetic acid, 50.0% ethanol and 0.5% glutaraldehyde) and after 72 hours of incubation, were stored in 70% ethanol. Prior to analyses, samples were dehydrated in increasing ethanol concentrations and completely dried by CO_2_ critical point with a Bal-Tec CPD 030 CO_2_ critical point dryer. Finally, samples were gold coated in a Bal-Tec SCD005 sputter coater and visualised in a Hitachi S-3500 N scanning electron microscope. Trichome counts were performed on the images obtained. Type-I, -III, -V, -VI and -VII as well as stomata were counted. Data are presented in boxplot as the mean ± standard deviation in boxplot graphs created using the online program BoxPlotR (http://shiny.chemgrid.org/boxplotr/). Genotype (WT vs. *hap*) as well as tissue source effect on trichome density were assessed by means of a Two-ways ANOVA test (Supplementary Table S3), whereas variance analyses were carried out using the least significance difference (LSD) post-hoc test of Fisher for mean comparison. A probability of *P* < 0,01 was considered statistically significant. Finally, data analysis from type I trichome density of transgenic lines was performed using a One-way ANOVA to test for genotype effect (*P* < 0,01) and for mean comparison a Dunnett’s test was used (Supplementary Table S4).

### Insect material and pest resistance assay

Moneymaker and *hap* mutant seedlings were transplanted to individual pots and kept under controlled humidity and light conditions in climate chambers (Aralab, Spain) with a programmed 18 hours light and 6 hours dark photoperiod, temperatures ranging from 25°C to 15°C during light and dark periods respectively and relative humidity set up in 65%. *Helicoverpa armigera* synchronic eggs were provided by Fendas (Almería, Spain) and maintained at 25°C until larvae hatched. When plants had developed four to five leaves, 24 hours-old *H. armigera* larvae were used for testing the WT and *hap* mutant pest resistance in a no-choice trial. Larvae were transferred to the apical fully expanded leaf of control and mutant plants with the aid of a fine paintbrush. Leaf damage percentage was recorded 15 days after larvae feeding by counting the number of damaged leaflets relative to the total of leaflets developed by the plants. Accounts were performed on images taken from each plant and genotype using a Canon Powershot SX20 digital camera.

### Genetic mapping of the *hairplus* mutation

With the aim to determine the chromosomic location of the *hap* mutation, a total number of 242 F_2_ plants derived from a cross between *S. pimpinellifolium* accession LA1589 and *hap* mutant were individually genotyped using codominant markers distributed along the genome as previously described [26]. Briefly, leaves from parents, F_1_ and F_2_ plants where frozen in liquid nitrogen and ground using a Retsch MM301 mixer mill shaker. Genomic DNA was isolated from approximately 300 mg of powdered leaf tissue using DNAzol® Reagent Kit (Invitrogen Life Technologies, USA) and following manufacturer’s instructions. DNA concentration was estimated by DNA quantitation using a Nanodrop 2000 spectrophotometer (ThermoFisher Scientific, USA) and by comparison with DNA standards after electrophoresis. Genetic linkage and distances were determined using JoinMap® 4 software.

### Whole genome sequencing and allele frequency analysis

DNA for whole genome sequencing was isolated using DNAzol® Reagent Kit as previously described. DNA isolation was performed individually for leaves from 14 F_2_ mutant plants as well as from WT plants, then a mutant and a WT pool were constructed using equal amounts of DNA from each plant. Illumina TruSeq DNA protocol was used for library generation and sequencing was performed in an Illumina HiSeq2000 platform (Illumina, Inc., USA) with 150 pb paired ends. Alignment of the obtained reads to the tomato genome reference sequence version 2.5 (ITAG2.4) was performed using Bowtie2 version 2–2.0.0-b5 with default parameters [49]. Picard version 1.65 was used to remove duplicated reads, whereas indels were realigned using GATK v2.2–8 under default settings [50]. Additionally, a variant calling was performed using GATK v2.2–8 and VCFtools [51] for variant filtering according to the following parameters: —min-alleles 2 —max-alleles 2 —min-meanDP 20 —max-meanDP 40. Afterwards, pileup from SAMtools 1.2 [52] was used for obtaining reference and non-reference allele counts for each position with the aim to determine the allele frequency ratio (i.e. non-reference allele counts / total allele counts) for bi-allelic variants. Finally, determination of the chromosomic region where the *hap* mutation is located was performed by plotting the average allele frequencies determined for each chromosome using a custom script in the R environment for statistical computing [53] that uses a sliding window and step size of 1000 and 100 variants, respectively.

### Transgenic analysis

A RNAi approach was used to obtain *HAP* (*Solyc10g077070*) silencing lines. A 185 pb sequence was amplified using the primers listed in Supplementary Table S5. The PCR product was cloned in sense and antisense orientation into the pKannibal vector and then digested with *NotI* restriction enzyme and cloned into the binary vector pART27 [54]. In addition, full-length coding sequence of *HAP* from WT plants (amplified with primers listed in Supplementary Table S5) was cloned into the pGWB402 vector driven by the CaMV 35S promoter for the generation of overexpression lines. CRISPR-Cas9 lines were also obtained following the protocol described by Vazquez-Vilar *et al.* (2016) [55]. sgRNA target sequence was designed within the coding sequence of *HAP* using the Breaking-Cas software [56] and the sgRNA selected was the one with the lowest off-target score. The construct contained the *A. thaliana* U626 promoter for constitutive expression of sgRNA, as well as the transcriptional unit for Cas9 expression in plants under the control of the CaMV 35S promoter. The kanamycin resistance gene was used as a selective marker. Finally, genetic transformation of all constructs was performed using *Agrobacterium tumefaciens* strain LBA4404 and T_0_ ploidy analysis was determined by flow cytometry as previously described [54, 57, 58]. Primers covering the sgRNA recognition site (Supplementary Table S5) were designed and T_0_ CRISPR-Cas9 lines were genotyped. PCR products were cloned into the pGEMT easy vector (Promega, USA) and eight clones per each PCR product were sequenced for further allele characterisation. Moreover, quantitative assessment of genome editing was performed using the program Tide (https://tide.deskgen.com/).

### RNA isolation and gene expression analysis

Total RNA was isolated from target tissues with TRIzol® Reagent (Invitrogen Life Technologies, USA) and following manufacturer’s protocol. DNA contamination was avoided by treatment with DNA-free™ DNA removal kit (Invitrogen Life Technologies, USA). RNA integrity was determined by denaturing agarose gel electrophoresis and quantitation was performed using a Nanodrop 2000 spectrophotometer (ThermoFisher Scientific, USA). First-strand cDNA synthesis was carried out with M-MuLV reverse transcriptase (ThermoFisher Scientific, USA), using a mixture of random hexamer and oligo(dT)_18_ primers. Gene expression analysis was conducted by qRT-PCR using the 7300 Real-Time PCR System (Applied Biosystems, ThermoFisher Scientific, USA) and SYBR Green PCR Master Mix (Applied Biosystems, ThermoFisher Scientific, USA). In all experiments, three biological replicates per genotype, each one from different plants grown in a random distribution in the same greenhouse, and two technical replicates were analysed [57]. Sequence of primers used for qRT-PCR has been previously described [11, 17, 19, 21, 22, 29] and those of the *hap* gene are shown in Supplementary Table S5. Raw data were analysed using the 7300 System Sequence Detection Software v1.2 (Applied Biosystems, ThermoFisher Scientific, USA). Sample normalization was performed for the housekeeping gene *UBIQUITIN3* and Ct calculation method [58] was carried out for quantitation of relative gene expression.

### RNA and methylation- sequencing

RNA isolation from inflorescence stems of Moneymaker and *hap* mutant plants was performed with TRIzol® Reagent (Invitrogen Life Technologies, USA), whereas DNA for whole genome bisulphite sequencing experiments was isolated from the same samples employing DNAzol® Reagent Kit (Invitrogen Life Technologies, USA) following manufacturer’s instructions. Samples consisted of three biological replicates per each genotype, each one from different plants grown in a random distribution in the same greenhouse, as previously described [57]. Preparation of libraries was performed following Illumina TruSeq RNA protocol and sequenced on the Illumina HiSeq2000 platform (Illumina, Inc., USA) with paired-end 150 pb. Quality control and adapter trimming of paired-end short reads were carried out by means of FastQC (https://www.bioinformatics.babraham.ac.uk/projects/fastqc/) and Trim-Galore (https://github.com/FelixKrueger/TrimGalore). Then, the filtered paired-end short reads were aligned to the reference genome (*S. lycopersicum* ITAG 2.50) by means of STAR. The Bioconductor R packages Rsamtools (http://bioconductor.org/packages/release/bioc/html/Rsamtools.html), GenomicFeatures and GenomicAlignments and SystemPipeR were used for transcript assembly and abundance estimation. Differentially expressed genes were identified by means of custom python scripts (https://github.com/cris12gm/rnaseqScripts). Genes with |FC| > 2 were selected in all mutant vs WT comparisons. RNAseq variant calling was carried out by means of bcftools mpileup and bcftools isec utilities [52]. The same samples used for RNAseq analysis were used for whole-genome bisulphite sequencing as previously described [59]. Finally, Differentially Methylated Cytosines (DMCs) analyses were performed by means of the logistic regression function implemented in *methylKit* [37] when replicates were available considering a *q*-value<0.01 and percent methylation difference larger than 25%. All raw sequencing data of the epigenome and transcriptome are available at NCBI under accession number PRJNA592298.

### HAP phylogenetic tree construction tools

Database search of HAP coding sequence was performed using Sol Genomics Network (https://solgenomics.net/), as well as Tair (https://www.arabidopsis.org/) for *A. thaliana* homologues. A phylogenetic tree was constructed by computing first a Multiple Sequence Comparison by Log-Expectation using MUSCLE and neighbour joining method using 10^3^ bootstrapping iterations.

### Histone methyltransferase activity assay

Full length open reading frame of HAP was amplified from the pGWB402 vector using the primers listed in Supplementary Table S5 and cloned into the pGEX4T-3 expression vector. The resulting plasmid was transformed into *E. coli* BL21-CodonPlus-RIL cells (Agilent) and protein expression assays were then performed as previously described [58]. Briefly, cells containing pGEX4T-3 vector were grown to an exponential phase and protein expression was induced by adding IPTG. Bacteria were then disrupted by sonication and protein purification was performed using GST-sepharose (Pharmacia) according to the manufacturer’s instructions. Finally, Histone H3 (K4, K9 and K27) methyltransferase activity was determined using HMT activity quantification assay kits (Abcam) following the manufacturer’s instructions.

## Supplementary Material

Web_Material_uhab015

## Data Availability

The datasets used and/or analysed during this study are available from the corresponding author on reasonable request. All sequence data were deposited in GenBank under SRA accession number PRJNA592298.
