# Supplementary material for: Functional characterization of the tomato HAIRPLUS gene reveals the implication of the epigenome in the control of glandular trichome formation
Source: Hortic Res. 2022 Jan 28;9:uhab015. doi: 10.1093/hr/uhab015 (PMC8795820; doi:10.1093/hr/uhab015)
Supplement: Web_Material_uhab015 [file web_material_uhab015.zip › Supplementary material.docx]

**Supplementary material**

**Functional characterization of the tomato *HAIRPLUS* gene reveals the implication of the epigenome in the control of glandular trichome formation**

**Running Title:**

***HAIRPLUS* controls glandular trichome formation**

Rocío Fonseca^1†^, Carmen Capel^1†^, Fernando J. Yuste-Lisbona^1^, Jorge L. Quispe^1^, Cristina Gómez-Martín^2,3^, Ricardo Lebrón^2,3^, Michael Hackenberg^2,3^, José L. Oliver^2,3^, Trinidad Angosto^1^, Rafael Lozano^1^, Juan Capel^1^.

*^1^Centro de Investigación en Agrosistemas Intensivos Mediterráneos y Biotecnología Agroalimentaria (CIAIMBITAL), Universidad de Almería, Carretera de Sacramento s/n, 04120-Almería, Spain*

*^2^Department of Genetics, Faculty of Science, University of Granada, Campus de Fuentenueva s/n, 18071-Granada, Spain*

*^3^Laboratory of Bioinformatics, Centro de Investigación Biomédica, PTS, Avda. del Conocimiento s/n,18100-Granada, Spain*

*^†^R. Fonseca and C. Capel should be considered joint first authors.*

**Correspondence author**

Juan Capel, Department of Biology and Geology, Centro de Investigación en Agrosistemas Intensivos Mediterráneos y Biotecnología Agroalimentaria (CIAIMBITAL), Universidad de Almería, Edif. CITE II-B, Carretera de Sacramento s/n, 04120 Almería, Spain

Email: jcapel@ual.es

**
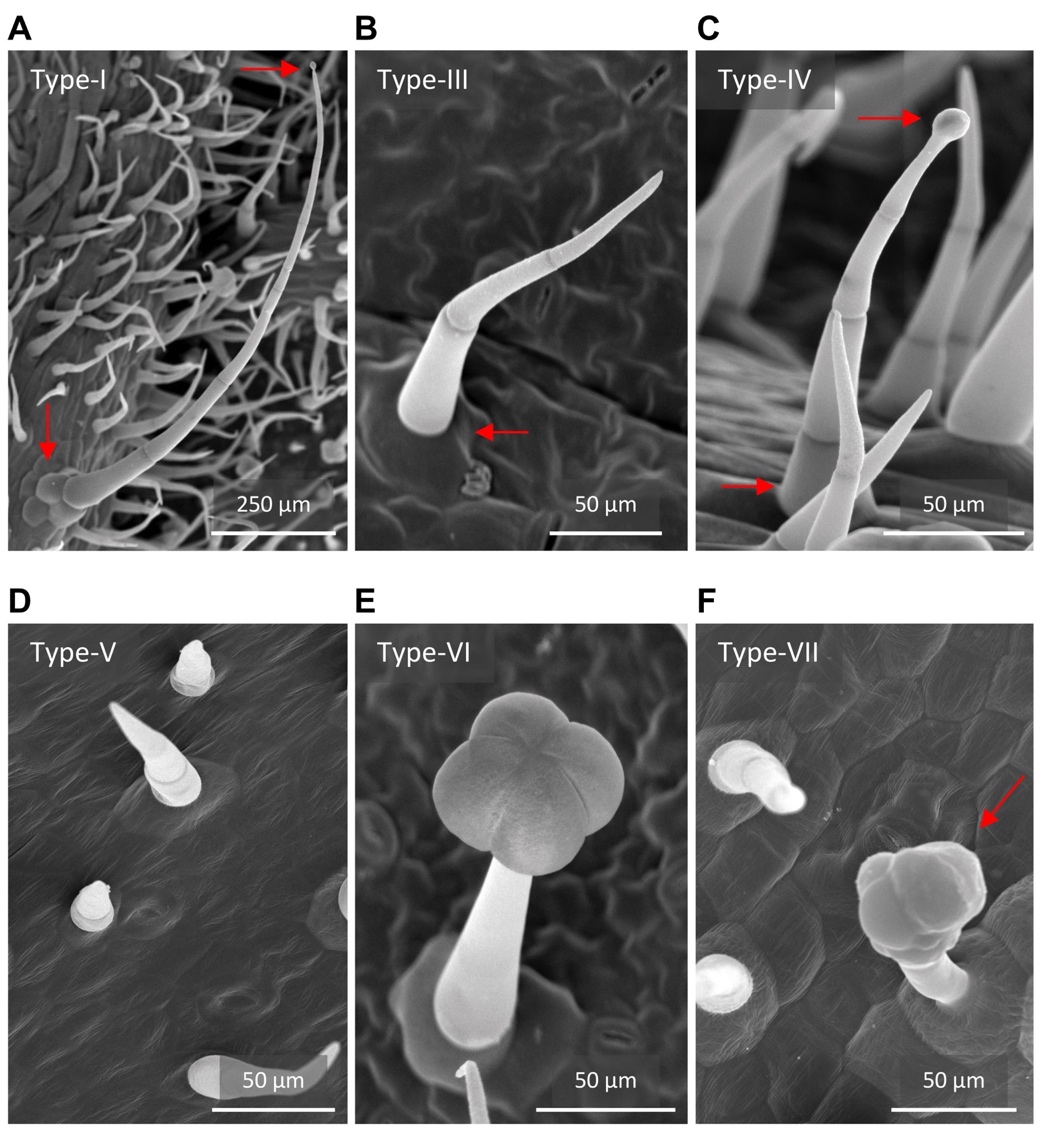
**

**Fig. S1.** Morphological overview of the trichome types of *S. lycopersicum.* (A) Type-I trichomes show a multicellular base and glandular head (red arrows). (B) Type-III non glandular and single celled base trichome (arrow). (C) Type-IV trichome with a unicellular base and glandular head (arrow). (D) Type-V trichomes. (E) Type-VI glandular trichome with a glandular head formed by four cells. (F) Type-VII glandular trichome with a glandular head formed by more than 4 cells (arrow).


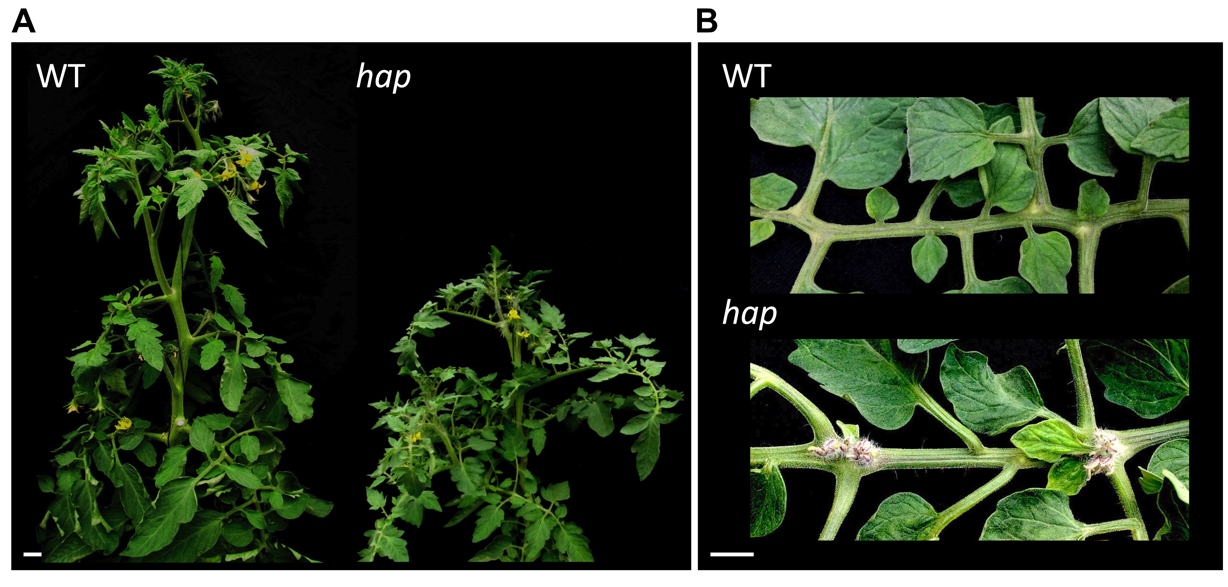


**Fig. S2.** Other aspects of the *hap* mutant phenotype. (A) Wild type plant and *hap* plant exhibiting lower growth. (B) Petiole of a WT plant and of a *hap* mutant plant showing developing plant shoots. All scale bars apply to 1 cm.


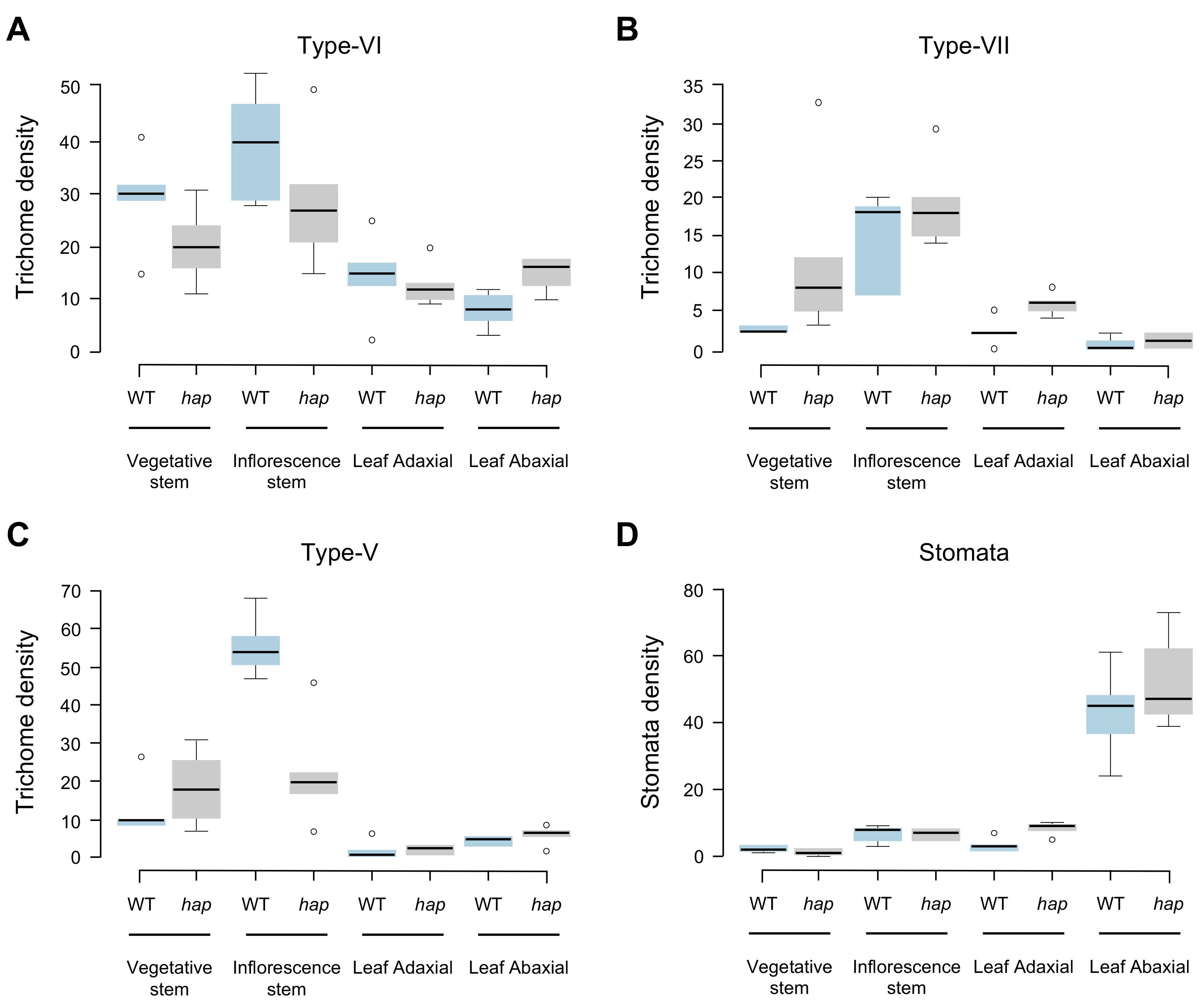


**Fig. S3.** Trichome and stomata density in main vegetative stem, inflorescence stem and leaves of WT and *hap* mutant plants. (A) Type-VI trichome density. (B) Type-VII trichome density. (C) Type-V trichomes density. (D) Stomata density. Center lines show the average values, whereas box limits indicate the 25th and 75th percentiles as determined by R software. Whiskers extend 1.5 times the interquartile range from the 25th and 75th percentiles and outliers are represented by dots. Genotype and tissue source effect on trichome density were assessed by means of a two-ways ANOVA analysis (*P* < 0.01). Statistical analyses were performed with Fischer´s LSD method (*P* = 0,05) and no significant differences were found.


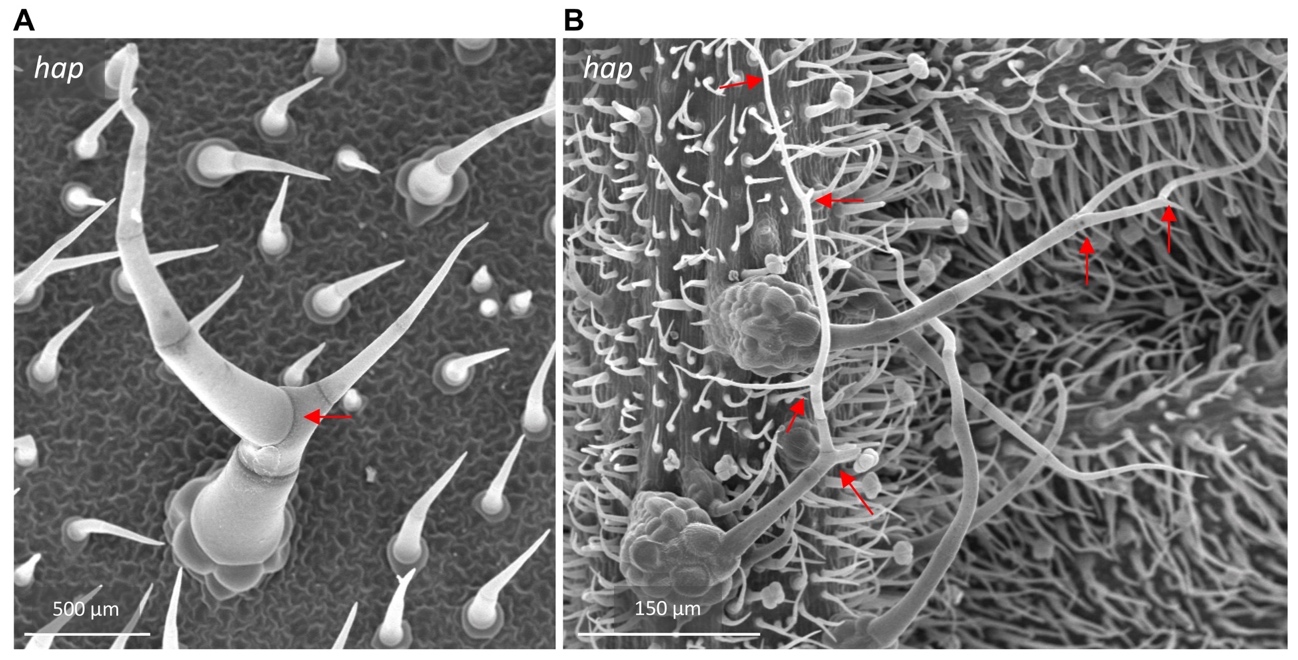


**Fig. S4.** Aberrant branching morphology observed in type-I trichomes of the *hap* mutant. (A) A branched type-I trichome developing in a young leaf of the *hap* mutant. (B) Branching of type-I trichomes is more evident in fully developed trichomes in older leaves, where two or more branches are produced. Red arrows point to each ramification observed.


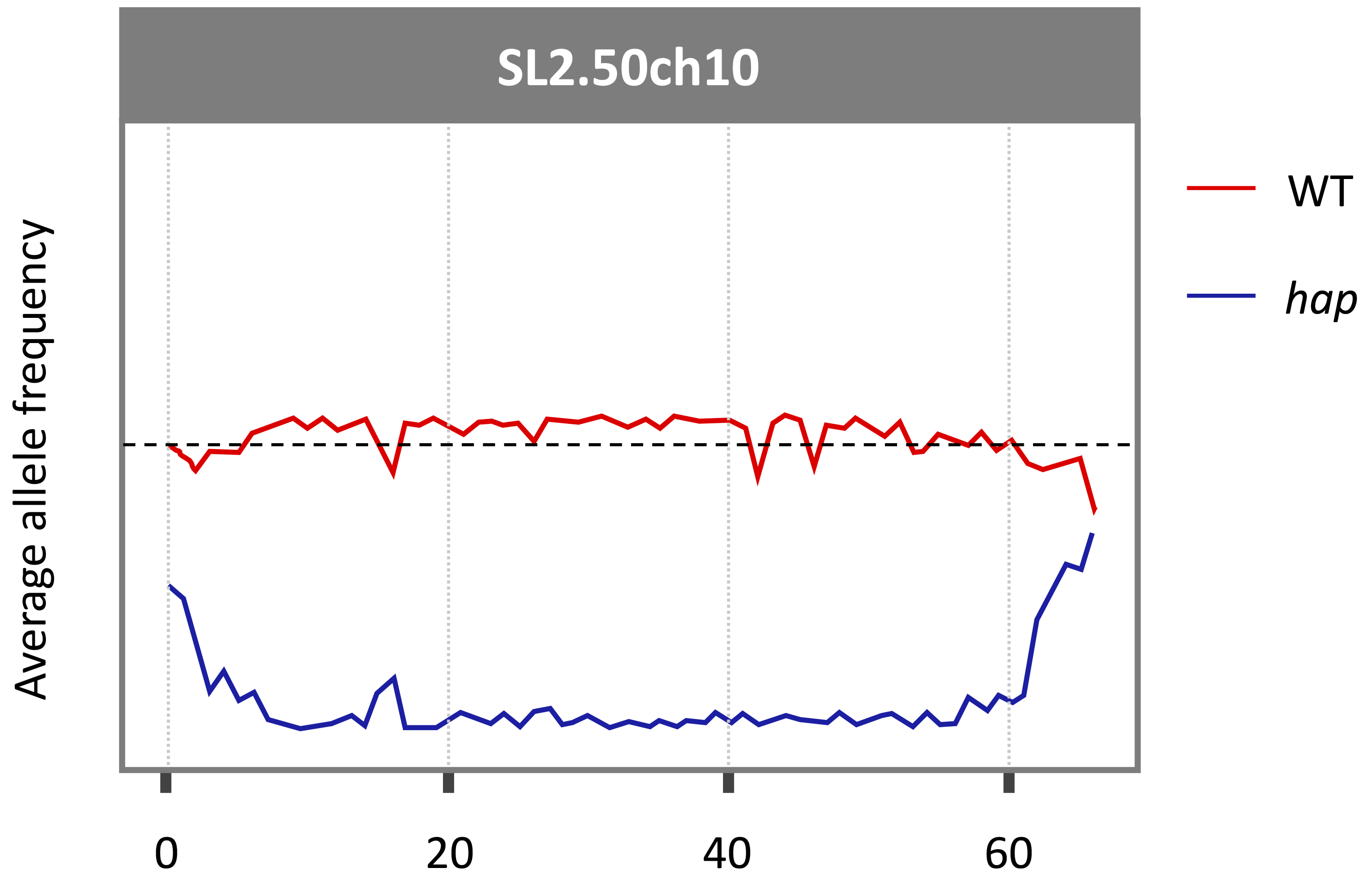


**Fig. S5.** Mapping by sequencing identification of *HAP*. Allele frequencies obtained from the sequencing of a DNA pool from 14 mutant plants (*hap*) compared with those observed in the sequence of a DNA pool from 14 wild type plants (WT), all of them F_2_ descendant from the cross between a *hap* mutant plant and a plant of *S. pimpinellifolium* accession LA1589. The centromeric region of chromosome 10 showed a drop in the average allele frequency indicating that *HAP* is located in this portion of chromosome 10.


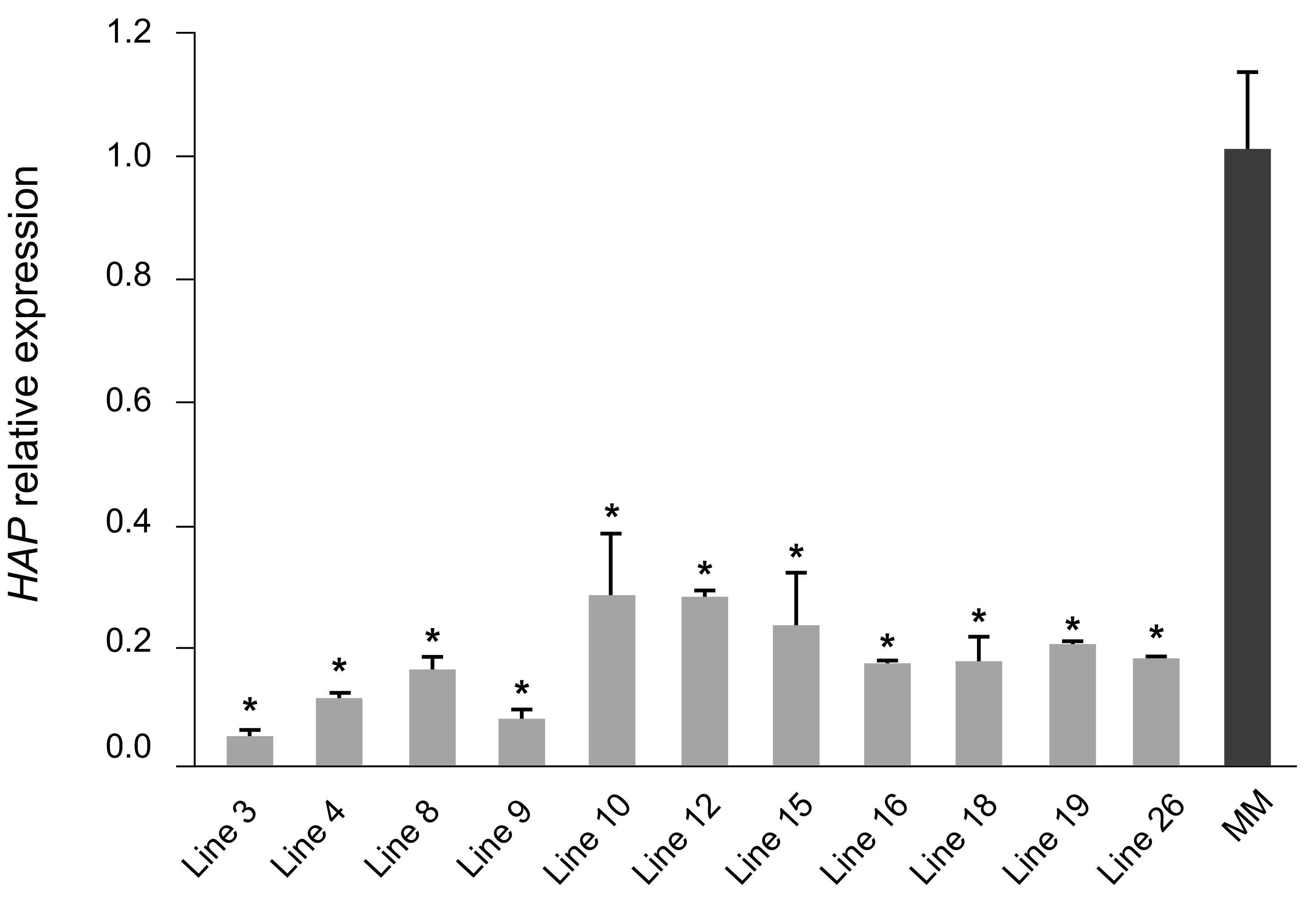


**Fig. S6.** Expression analysis of *HAP* in RNAi silencing lines. Expression in *HAP* silencing lines (RNAi) decreased 4- to 25-fold (relative expression levels of 0.27 in line 12 and 0.04 in line 13, respectively) when compared to WT plants. Asterisks account for significant differences at *P* < 0.05.


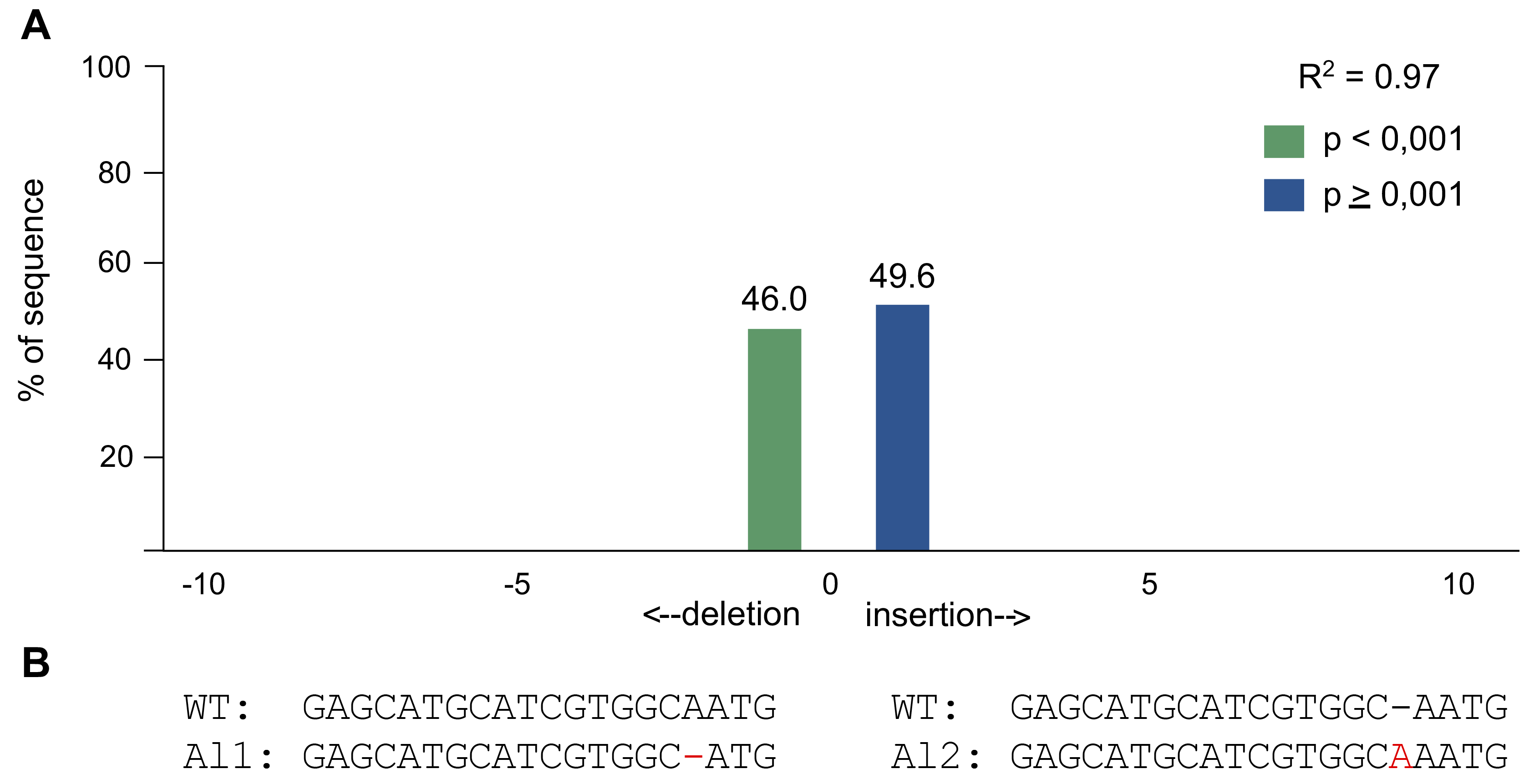


**Fig. S7.** Allele characterisation in the CRISPR-Cas9 knock-down line CRISPR1. (A) Quantitative assessment of genome editing performed with the program Tide (https://tide.deskgen.com/) confirmed that it is a biallelic line. (B) Allele sequence of the target sgRNA in the CRISPR1 line confirmed the presence of novel mutant *hap* alleles.


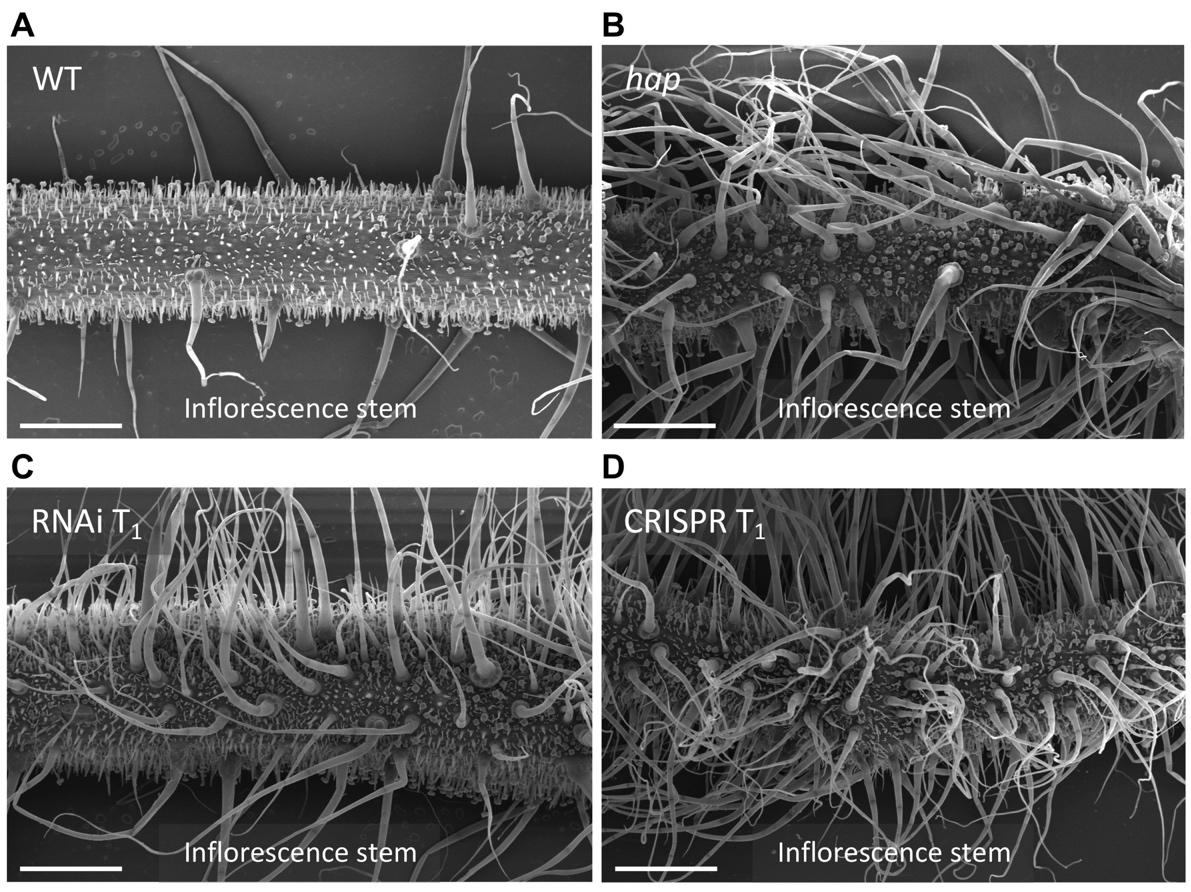


**Fig. S8.** *HAP* controls type-I trichome density. (A) Inflorescence stem of a WT plant. (B) Inflorescence stem of a *hap* mutant plant. (C) Inflorescence stem of a *HAP* RNAi silencing line descendant. (D) Inflorescence stem of a non-transgenic but homozygous for the CRISPR-Cas *hap* knock-out allele plant.


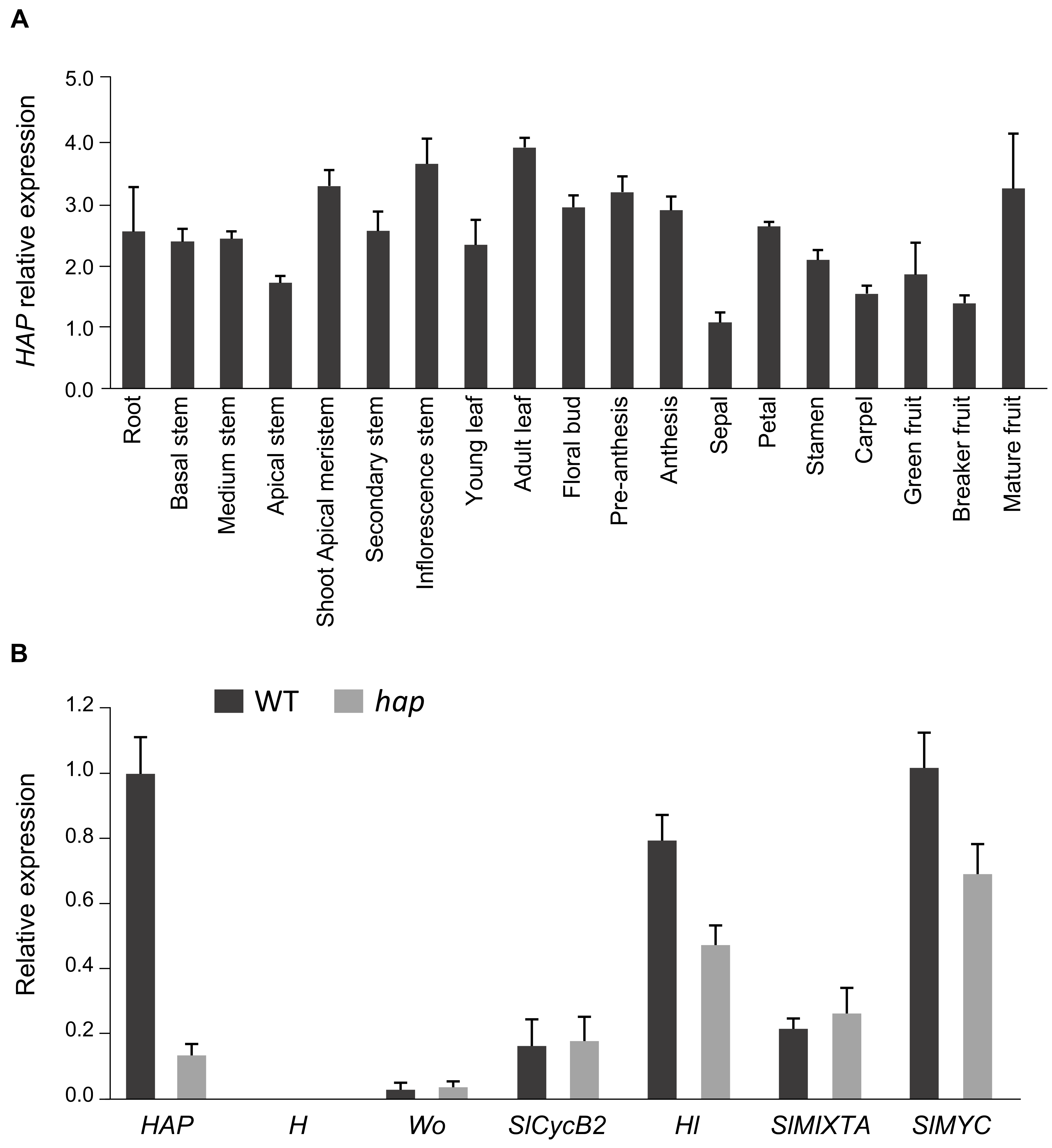


**Fig. S9.** Expression pattern of *HAP* and changes of expression in *hap.* (A) Relative expression was determined by real time RT-PCR using three biological replicates of different tissues from WT plants. (B) Expression analysis performed in WT and *hap* mutant plants demonstrates that *HAP* is repressed in *hap* mutant. None of the analysed genes *Hair* (*H*), *Woolly* (*Wo*), *SlCycB2*, *Hairless* (*Hl*), *SlMixta-like* (*SlMixta*) and *SlMYC1* showed significative differences in their relative expression in *hap* mutant plants.

**
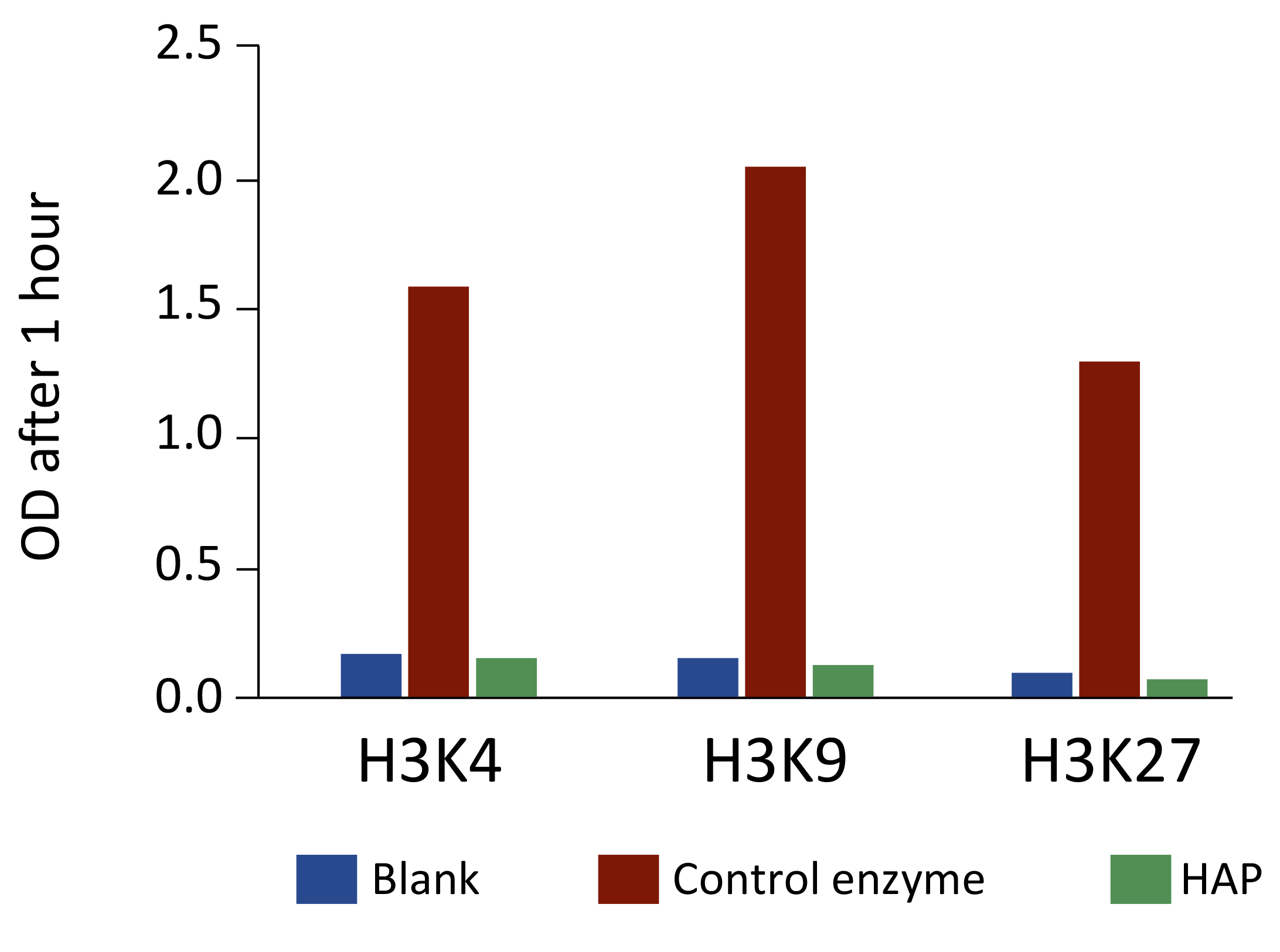
**

**Fig. S10.** HAP does not display HMT activity *in vitro.* HMT assay using different H3 lysine residues as substrates. Blank = no enzyme added.


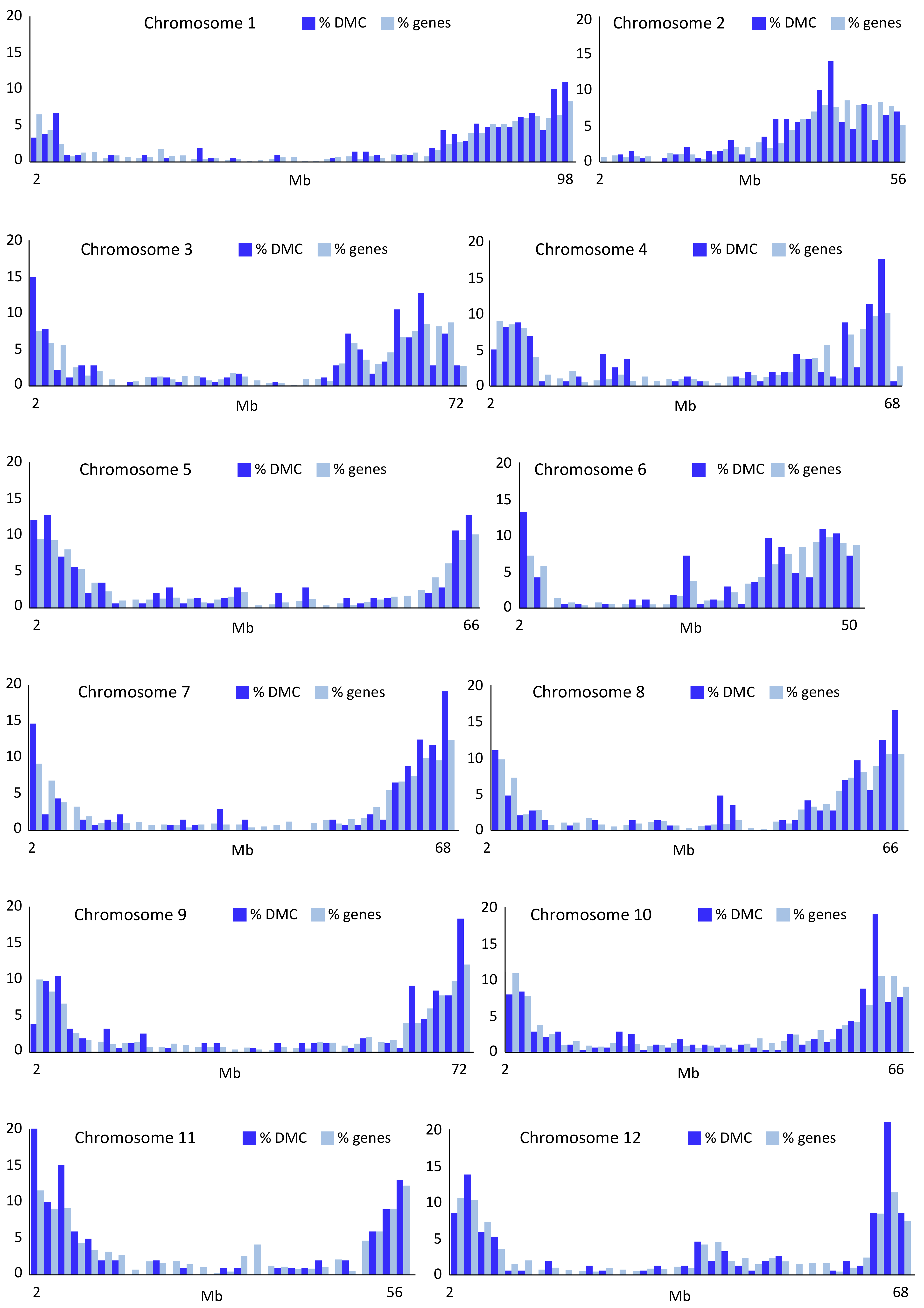


**Fig. S11.** Distribution in the 12 tomato chromosomes of the DMCs caused by *HAP’s* loss of function. The dark blue histograms represent the percentage of DMCs every 2 Mb whereas the light blue histograms show the density of genes.

**
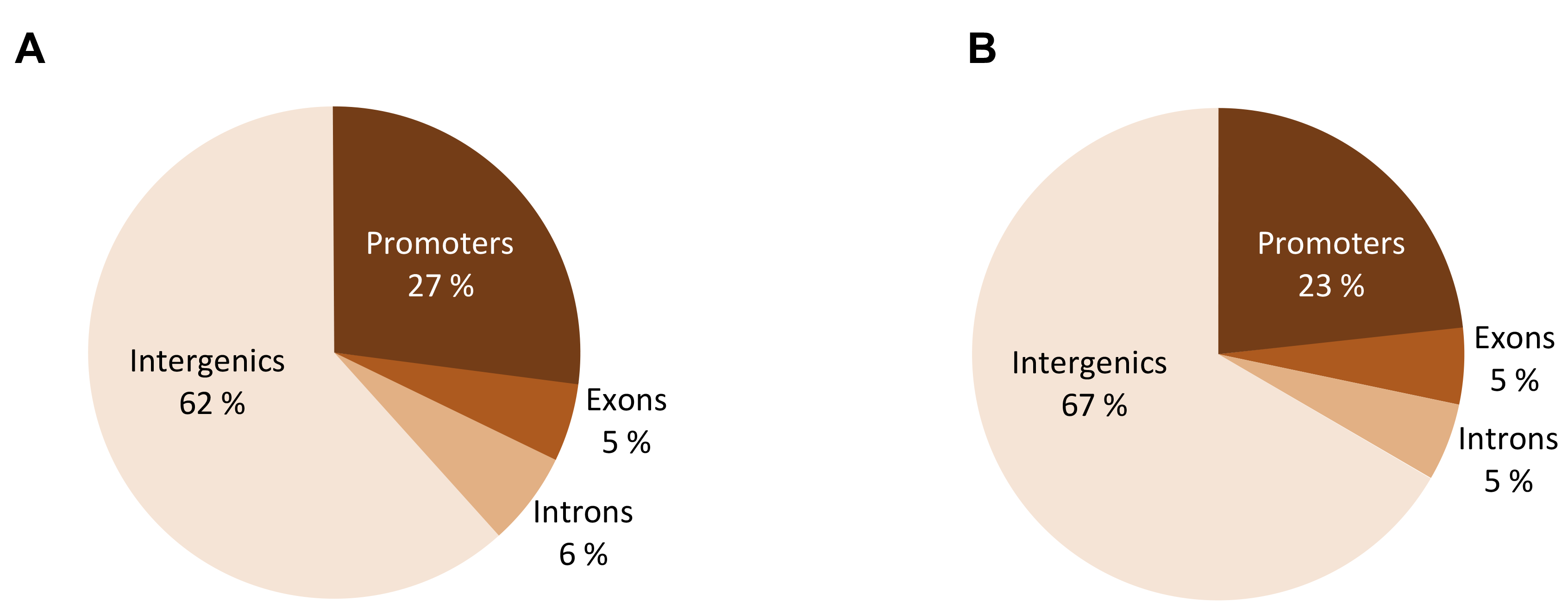
**

**Fig. S12.** Location of DMCs caused by *HAP’s* loss of function. (A) Location of hypomethylated CpG, (B) differential location of hypermethylated CpG.


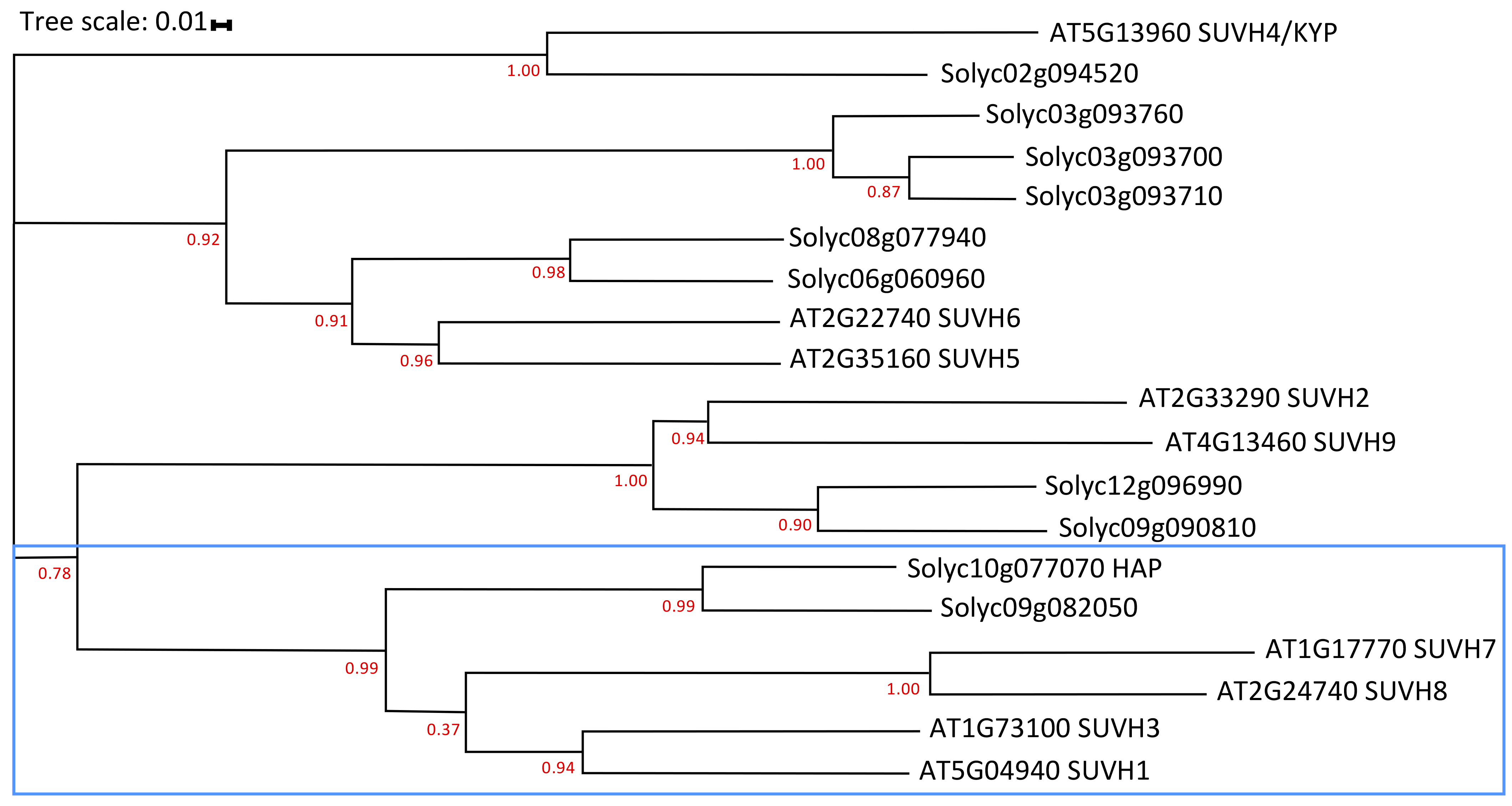


**Fig. S13.** Phylogenetic relationships between *Arabidopsis* and tomato SET domain containing proteins. The tree was constructed using the alignment program MUSCLE (https://www.ebi.ac.uk/Tools/msa/muscle/). Bootstrap values are represented in red. Outlined by blue box is the clade that groups HAP and its most closely related proteins.

AT5G13960_SUVH4/KYP ------------------------------------------------------------

Solyc02g094520 ------------------------------------------------------------

Solyc03g093760 --------------------MVDTESPSTFKRLKIH------------------------

Solyc03g093700 MSVLAETSPSEMLGKKKLHQMVDTKSPSTFKRVKVD------------------------

Solyc03g093710 -----------MLGKKKLHQMVDTKSPSTVKRLKVD------------------------

Solyc08g077940 -----MVSF---------------------------------------------------

Solyc06g060960 -----MIYWTELDINSTIWMVMIHIENEFFCRMISIILNTDLLGQRRNRYQMSSFQFQDS

AT2G22740_SUVH6 ---------------MEMGVMENLMVHTEISKVK--------------------------

AT2G35160_SUVH5 ---------------------MVHSESSILSSLR--------------------------

AT2G33290_SUVH2 ------------------------------------------------------------

AT4G13460_SUVH9 ------------------------------------------------------------

Solyc12g096990 ------------------------------------------------------------

Solyc09g090810 MYFINKILKIYKISNFTCISTKKRKVKFIILILLL-------------------------

AT1G17770_SUVH7 ------------------------------------------------------------

AT2G24740_SUVH8 ------------------------------------------------------------

Solyc10g077070_HAP ------------------------------------------------------------

Solyc09g082050 ------------------------------------------------------------

AT1G73100_SUVH3 ------------------------------------------------------------

AT5G04940_SUVH1 ------------------------------------------------------------

AT5G13960_SUVH4/KYP -----------------MAGKRKRANAP----DQTERRSSVRVQKVRQKALDEKAR----

Solyc02g094520 ---------MVVPCVAELSDPVNDAMVPRR--CSARIKKLKSEQEAQR-ERESQRVRCRS

Solyc03g093760 --ATRNFPENCGPFVCQNNGSRK-IYPEFP--SNTKRVKVDSRRSFPE-NCGPQKRD---

Solyc03g093700 --ATRNFPENCGSFVYQNNGSKD-IYPEFR--SNSKRVKVNSTRSFPK-NCGPCVPEKKK

Solyc03g093710 --ATRNFPENCGPFVGENDGTGD-KYPEFP--SATKPVKVETTRNYPE-NCGPCVLQKKN

Solyc08g077940 --SNDGLSDQCVKKRSSVNGYHL-LDSGTM--SKHKVRIVCGEQDLPP-GCSRNAPKVDL

Solyc06g060960 NVSKDGLSNKSVKKRLLENGCHS-SYLGII--PKYKIRKVSAVRDFPP-GCGRTSLKVDL

AT2G22740_SUVH6 --SQSNGEVEKRGVSVLENGGVCKLDRMSG--LKFKRRKVFAVRDFPP-GCGSRAMEVKI

AT2G35160_SUVH5 --GGDGGGIPCSKDELAINGSYTDPMGR----RKSKRFKVAAESEFSP-DFGSITRQLRS

AT2G33290_SUVH2 ---------------------------------------MSTLLPFPDLNLMPDSQSSTA

AT4G13460_SUVH9 ------------------MGSSHIPLDPSLNPSPSLIPKLEPVTESTQ-NLAFQLP----

Solyc12g096990 ----------------MEMGSVV-GLGDVN--FSTEPKTPTPTMIFPK-I----------

Solyc09g090810 --HLFTLSPYEHTCHFCKTSKLCSLFDFLK--TQNPIFDLGSLVPFQDLNLQPESTNFTS

AT1G17770_SUVH7 -------------------------------MDKSIPIKAIPVACVRP-DLVDDVTKNTS

AT2G24740_SUVH8 -----MVSTPPTLLMLFDDGDAGPSTGLVHREKSDAVNEEAHATSVPP-HAPPQTLWLLD

Solyc10g077070_HAP ----------------MEQGFGSDSVPPAGPIDKSKVLDVKPLRCLVP-VFPS-------

Solyc09g082050 ----------------MEQGFGSDPSGSTI--DKTRVLDVKPLRCLSP-VFPSASE----

AT1G73100_SUVH3 --------------MQGVPGFNTVPNPNHY--DKSIVLDIKPLRSLKP-VFPN-------

AT5G04940_SUVH1 --------------MERNGGHYT---------DKTRVLDIKPLRTLRP-VFPS-------

AT5G13960_SUVH4/KYP ---------------------LVQERVKLL------------------------------

Solyc02g094520 ----------NDDSVLGKKT-KVYKKSKLV------------------------------

Solyc03g093760 ----------GSDTQCSVDA-DNNSCSEVE------------------------------

Solyc03g093700 ----------GSDTPCSVDS-EIKSCSDVDMNVVESAEPLSVFEP---------------

Solyc03g093710 ----------GCDTQSSANV-DIGSCSEVEMDVVELGDPLSVFVPK--------------

Solyc08g077940 ----------NQNENAMVSI-S-ENMADTLVAHGDNGPNTGVEFCSVEVAS---------

Solyc06g060960 ----------NHVQNAEVST-NIEDMTNIILVDGVKETNIEVKSQSVEVVNDLINLENQE

AT2G22740_SUVH6 ----------ACENGN-VVE-DVKVVESLV------------------------------

AT2G35160_SUVH5 RRMQKEFTVETYETRNVSDVCVLSSQADVELIPGEIVAERDSF-----------------

AT2G33290_SUVH2 ----------GTTAGDTVVTGKLEVKSEPI------------------------------

AT4G13460_SUVH9 ----------NTNPQALISS-AVSDFNEAT------------------------------

Solyc12g096990 -----------------------EPKLEPL------------------------------

Solyc09g090810 ----------STTPNPRIIP-KIEPKLEPL------------------------------

AT1G17770_SUVH7 ----------TIPTMVSPVLTNMPSATSPL------------------------------

AT2G24740_SUVH8 ----------NFNIEDSYDR-DAGPSTGPV------------------------------

Solyc10g077070_HAP --------------PNGMAS-GTTPQPSPF------------------------------

Solyc09g082050 -----------------MSS-ITTPQPSPF------------------------------

AT1G73100_SUVH3 -----------------------GNQGPPF------------------------------

AT5G04940_SUVH1 -----------------------GNQAPPF------------------------------

AT5G13960_SUVH4/KYP ------------------------------SDRKSEICVDDTELHEKEEENVDGSP----

Solyc02g094520 ----------------------------TPSQAQTQAPNNDVTVATVDNDDVTITNVG--

Solyc03g093760 -------------------------------SAESCNFEATGNQPLKLKEENVIYDESTQ

Solyc03g093700 ------------EDDLAATVVCPKEAGDSSHQNTSCQPANGNQQHEVLVNLVLQNPSID-

Solyc03g093710 ----------DMQFDLDATGVCEEEGGDSSHLNTSCQPVTNGNQVLTTKEVNLMYDDSTQ

Solyc08g077940 ----------ARTTNVIENGLEEPTSHDKSLRFELSKDHKNSEMSLLKKAKVIGYDELGT

Solyc06g060960 NVDRLAGEVMATNMSAIANGVGEKISDEKSTGFELPKDLKTSEMELSKETEDIQNDTSVK

AT2G22740_SUVH6 ---------------KEEESLGQRDASENVSDIRMAEPVEVQPLRICLPGGDVVRDLSVT

AT2G35160_SUVH5 ------------KSVDCNDMSVGLTEGAESLGVNMQEPMKDRNMPENTSEQNMVEVHPPS

AT2G33290_SUVH2 --------------------------------EEWQTPPSSTSDQSANTDLIAEFIRISE

AT4G13460_SUVH9 ---------------------------DFSSDYNTVAESARSAFAQRLQRHDDVAVLDSL

Solyc12g096990 -------------------DEFTPQSMNPNSNFSYNSGFRNTTTPQQQQLNATSSQTPSS

Solyc09g090810 ------------DEYTQADLQTPAFFSNPSPNFNTSSGSAFRRNPQLATHEADSQSPSSI

AT1G17770_SUVH7 -------------------LMVPPLRTIWPSNKEWYDGDAGPSSTGPIKREASDNTNDTA

AT2G24740_SUVH8 --------------------HRERSDAVNEEAHATSIPPHAPPQTLWLLDNFNIEDSYDR

Solyc10g077070_HAP --------------------VCVPPSGPFPPGVSPFYPFLSPNESGRSAENQDG------

Solyc09g082050 --------------------LCITPTGPFPSGVTPIFPFLSPDEPVRMGESSQQTPNQVP

AT1G73100_SUVH3 --------------------VGCPPFGPSSSEYSSFFPFGAQQPTHDTPDLNQT------

AT5G04940_SUVH1 --------------------VCAPPFGPFPPGFSSFYPFSSSQANQHTPDLNQAQYPPQH

AT5G13960_SUVH4/KYP ------------------------------------------------------------

Solyc02g094520 ------------------------------------------------------------

Solyc03g093760 HHQVQKQ-----------------------------------------------------

Solyc03g093700 ------------------------------------------------------------

Solyc03g093710 LNEVLVNQILQKTSTD--------------------------------------------

Solyc08g077940 EVDVARHFFLVENVIGMYKDHVLHPGSMTDRVIPVCDSKTLSLPQCQIKNGSVEDNISPL

Solyc06g060960 EVD-EQGLPLVESING---------GHMTQKLISVMEHTSTS------------------

AT2G22740_SUVH6 ------------------------------------------------------------

AT2G35160_SUVH5 ISLPEEDM----------------------------------------------------

AT2G33290_SUVH2 LF----------------------------------------------------------

AT4G13460_SUVH9 ------------------------------------------------------------

Solyc12g096990 IEAGV-------------------------------------------------------

Solyc09g090810 IPEVPPGCDRNNVYV---------------------------------------------

AT1G17770_SUVH7 HN----------------------------------------------------------

AT2G24740_SUVH8 DAGPSTSPIDREASHEVNEDAHA-------------------------------------

Solyc10g077070_HAP ------------------------------------------------------------

Solyc09g082050 NQGT--------------------------------------------------------

AT1G73100_SUVH3 ------------------------------------------------------------

AT5G04940_SUVH1 QQPQNPPPVYQQQPP---------------------------------------------

AT5G13960_SUVH4/KYP -KRRSPPKLTAMQKGKQKLSVSLNG-----------------------------------

Solyc02g094520 -APIDCTDHPVPENSLNPQLSGNGT-----------------------------------

Solyc03g093760 -STDTFDWFIKDEPIENGPAIVSQE-------NLIDCQNDEPSKETCQSVHREEVSDDES

Solyc03g093700 -SGNTCDWFIKSEPIENEPELPAIV-----SQENLIQGRDEPSKETSKRVHYGEVPYDEY

Solyc03g093710 -TGNTCDWFINGDPIENGPELPSEE-------------------------TNKGFQYKEV

Solyc08g077940 PKKKYCRRGVFAVRDFPPFCGRNAPKSTKLDLLGGNEASKRAILLNKGVTENEVI--ETS

Solyc06g060960 PKNKYRKRRVSAVRDFPPFCGTKVPKSTEQNCFGVTEESKDVAGFGKAVTRNEVI--ETL

AT2G22740_SUVH6 -AGDECSNSEQIVAGSGVSSSSGTE----------------------NIVRDIVVYADES

AT2G35160_SUVH5 -MGSVCRKSITGTKELHGRTISVGR--------------DLSPNMGSKFSKNGKTAKRSI

AT2G33290_SUVH2 -RSAFKPLQVKGLDGVSVYGLDSGA--------------------------------IVA

AT4G13460_SUVH9 -TGAIVPVEENPEPEPNPYSTSDSS-----------------------------------

Solyc12g096990 -HSEYNRISELFQTAFAQSVQRDGD-----------------------------------

Solyc09g090810 -YSEYNRISEMFKEAFTEKMQRYGD-----------------------VEVVGNQNQDSV

AT1G17770_SUVH7 -TFAPPPEMVIPLITIRPSDDSSNY-------------------------SCDAGAGPST

AT2G24740_SUVH8 -TSAPPHVMVSPLQNRRPFDQFNNQ-------------------------PYDASAGPST

Solyc10g077070_HAP -LGFGTPISPVPLNSFRTPAANGDT-----------------------GPRRPGRPRASN

Solyc09g082050 -FGFGQPISPIPVNSFGNQTANGSS-------------------------------GHVN

AT1G73100_SUVH3 -QNTPIPSFVPPLRSYRTPTKTNGP--------------------------------SSS

AT5G04940_SUVH1 -QHASEPSLVTPLRSFRSPDVSNGN-----------------------------------

AT5G13960_SUVH4/KYP ------------------------------------------------------------

Solyc02g094520 ------------------------------------------------------------

Solyc03g093760 RSWVDDDDISILTCSEWNSLTSALKDGKKGGKEGEIIHK---------------CSDILE

Solyc03g093700 RSRVDNDEICILSCSESNSLKSGLKTLSAGKKGGK-------------------GEIVQE

Solyc03g093710 ADDESTSRVDNSSCSQSNSQNSGLKTPSASKKGGK-------------------GEIVQE

Solyc08g077940 KNVMDTGTLSLGLTASREADSWSKTEVTGSKCSLIERAT-VRVEDPEDVQDNYVRRSQLE

Solyc06g060960 REVTETGALPEKLIGSEDADSLKDRDVSSPKDRQLEQITMVRTEEQEGVQCDYDGRSQVE

AT2G22740_SUVH6 SLGMDNLDQTQPLEIEMSDVAVAKPRLVA-------------------------GRKKAK

AT2G35160_SUVH5 SVEEENLVLEKSDSGDHLGPSPEVLELEKSE-----------------------VWIITD

AT2G33290_SUVH2 VPEKENRELIEPPPGFKDNRV---------------------------------STVVVS

AT4G13460_SUVH9 ------------------------------------------------------PSVATQ

Solyc12g096990 VEANEDLGCRAIVPVSNGSQV---------------------------------SDIVIT

Solyc09g090810 DVVMEDADARAIVPVSNNDTQV--------------------------------AEVVVA

AT1G17770_SUVH7 GPVKRGRGRPKGSKNST-------------------------------------PTEPKK

AT2G24740_SUVH8 GPGKRGRGRPKGSKNGSRKPKKPKAYDNNSTDASAGP-----------------SSGLGK

Solyc10g077070_HAP GLAAEDDDSQNHSDQFGSGYSGHANDVEDTSTGKK-------------------RGRPRK

Solyc09g082050 NVGDSGSGKK--------------------------------------------KGGPKK

AT1G73100_SUVH3 SGTKRGVGRPKGTTSVKKKE----------------------------------KKTVAN

AT5G04940_SUVH1 -AELEGSTVK--------------------------------------------RRIPKK

AT5G13960_SUVH4/KYP ------------------------------------------------------------

Solyc02g094520 ------------------------------------------------------------

Solyc03g093760 DFKPLPDIIRPEQ----------------------------QYE----------------

Solyc03g093700 EAVSSPEPLHKCNV---------------------------IFE----------------

Solyc03g093710 EAVKCPEPLHKCKV---------------------------IFE----------------

Solyc08g077940 RTVMLPETMTKKERDDTGKFLLKESIVYSRNEREKATTARHGFGSGDK------------

Solyc06g060960 RTVVMPEIMTKKG-SDAGP-VGKETLVYSENEREKLTSASSALGSGNEKQITKGAKPSGA

AT2G22740_SUVH6 KGIACHSSLKVVSR---------------------------EFG----------------

AT2G35160_SUVH5 KGVVMPSPVKPSEKRNG------------------------DYG----------------

AT2G33290_SUVH2 PKFERPRELARIA----------------------------ILG----------------

AT4G13460_SUVH9 RPRPQPRSSELVRIT--------------------------DVG----------------

Solyc12g096990 RRKYEKRSSELVRVT--------------------------DLK----------------

Solyc09g090810 RRKYQQRSSELVRVT--------------------------DLK----------------

AT1G17770_SUVH7 PKVYDPNSLKVTSRG--------------------------NFD----------------

AT2G24740_SUVH8 RRCGRPKGLKNRSRKPKKP----------------------KADDP--------------

Solyc10g077070_HAP TRLGQPSSGNPATPPIEV-----------------------DVD----------------

Solyc09g082050 PRKVPPENVVEV-----------------------------DVE----------------

AT1G73100_SUVH3 EPNLDVQVVKKFSS---------------------------DFD----------------

AT5G04940_SUVH1 RPISRPENM--------------------------------NFE----------------

AT5G13960_SUVH4/KYP ------------------------------------------------------------

Solyc02g094520 ------------------------------------------------------------

Solyc03g093760 ------------------------------------------------------------

Solyc03g093700 ------------------------------------------------------------

Solyc03g093710 ------------------------------------------------------------

Solyc08g077940 ------------------ITKPVVHGLMDERCSPWRQKKQTPRQIVQGLMAETNKDWRQK

Solyc06g060960 RKQGKQKSLDDPVSGNEIVVSQVESHLTKTAVNAFGSGHEIVKPIVQGLMAKPCCPWRQG

AT2G22740_SUVH6 ------------------------------------------------------------

AT2G35160_SUVH5 ------------------------------------------------------------

AT2G33290_SUVH2 ------------------------------------------------------------

AT4G13460_SUVH9 ------------------------------------------------------------

Solyc12g096990 ------------------------------------------------------------

Solyc09g090810 ------------------------------------------------------------

AT1G17770_SUVH7 ------------------------------------------------------------

AT2G24740_SUVH8 ------------------------------------------------------------

Solyc10g077070_HAP ------------------------------------------------------------

Solyc09g082050 ------------------------------------------------------------

AT1G73100_SUVH3 ------------------------------------------------------------

AT5G04940_SUVH1 ------------------------------------------------------------

AT5G13960_SUVH4/KYP ------------------------------------------------------------

Solyc02g094520 ------------------------------------------------------------

Solyc03g093760 ----------------SVFMKKQMDLGVPQEN----------------------------

Solyc03g093700 --------------DESVVMKNQIVLGVSQED----------------------------

Solyc03g093710 --------------HESVVRKKQIDIGVSPED----------------------------

Solyc08g077940 EQTRLDGLMSRNQVPKPSMYRQRMSVVVARKSIPKPKFPETLFGRSRSGFVGEAVPEYPS

Solyc06g060960 EPTSLD---CGNQVEKDDFSGRKKAKAVTRKSNPRGKKKSVTLGEATDGLSSALVVFNDK

AT2G22740_SUVH6 --------------EGSRKKKSKKNLYWRDRE----------------------------

AT2G35160_SUVH5 ---------------EGSMRKNSERVALD-------------------------------

AT2G33290_SUVH2 ------------------------------------------------------------

AT4G13460_SUVH9 ------------------------------------------------------------

Solyc12g096990 ------------------------------------------------------------

Solyc09g090810 ------------------------------------------------------------

AT1G17770_SUVH7 ------------------------------------------------------------

AT2G24740_SUVH8 ------------------------------------------------------------

Solyc10g077070_HAP ------------------------------------------------------------

Solyc09g082050 ------------------------------------------------------------

AT1G73100_SUVH3 ------------------------------------------------------------

AT5G04940_SUVH1 ------------------------------------------------------------

AT5G13960_SUVH4/KYP -------------------------------------------KDVNLEPHLKVTKCLRL

Solyc02g094520 ----------------------------------------------EKSSHARVTETLRI

Solyc03g093760 -------------SRNSAVMCGVSGHGFST-------------EYEHIHEVKQVRKTLKL

Solyc03g093700 -------------LRNSVVMCNVSGNGLLT-------------EHEHIQKVKEVRETLKL

Solyc03g093710 -------------LRNSDVFCGASGNGLLM-------------EHENIQKVKEVKETLKL

Solyc08g077940 SP---FSKNDGIRNLNCEAQPKDSPIGQKKCEFDETRPPFGPKSSSRCDARSKVLETLRL

Solyc06g060960 GPGLWATSNDGACSLNREAVHEDSPVRRGQCDFDVTLPPFGPNSSSHGDARTKVRETLRL

AT2G22740_SUVH6 -------------SLDSPEQLRILGVGTSSG------------SSSGDSSRNKVKETLRL

AT2G35160_SUVH5 -------------KKRLASKFRLSNGGLPSC------------SSSGDSARYKVKETMRL

AT2G33290_SUVH2 -------------------------------------------HEQRKELRQVMKRTRMT

AT4G13460_SUVH9 -------------------------------------------PESERQFREHVRKTRMI

Solyc12g096990 -------------------------------------------PEDVRYFRDLIRKTRML

Solyc09g090810 -------------------------------------------VEDQLYFREAVRKTRML

AT1G17770_SUVH7 -------------------------SEITE-------------AETETGNQEIVDSVMMR

AT2G24740_SUVH8 -------------NSKMVISCPDFDSRITE-------------AERESGNQEIVDSILMR

Solyc10g077070_HAP -------------PLLNQLLASFKLVEIDQ-------------VKKADGDKELSGRILLV

Solyc09g082050 -------------PLLNQLLMSFKLVDLDQ-------------AKKADGDKEVVRRILLV

AT1G73100_SUVH3 -------------------------SGISA-------------AEREDGNAYLVSSVLMR

AT5G04940_SUVH1 -------------------------SGINV-------------ADRENGNRELVLSVLMR

AT5G13960_SUVH4/KYP FNKQYLLCVQAKL-----------------------------SRPDLKGVTEMIKAKAIL

Solyc02g094520 FNKHYLHFVQEEEIRCGRAQADQKTKKHSKSKEAEDDGKRSSKRPDLKAISKMISEKEVL

Solyc03g093760 FDDVYTKLLQEDK-----------------AENPEGRS---KRKIHIEAAMTLKNQKKWV

Solyc03g093700 FDDEYTKLLLEDR-----------------AEKHEGGP---KRSIHIEAAMALKKQKKWV

Solyc03g093710 FDDEYTKLLQEDK-----------------AKKHEGRS---KRRIHIEAAMNLKKQKKWV

Solyc08g077940 FQSHFRKILQGEE-------------SMSRSAGVNAKQKDKIRRIDLQAAKLVKDKGKQV

Solyc06g060960 FQGICRKLLQGEE---------------SKSKPEEAKSKQGPNRIDLHAAKIIKEKGKEV

AT2G22740_SUVH6 FHGVCRKILQEDE-----------------AKPEDQRRKGKGLRIDFEASTILKRNGKFL

AT2G35160_SUVH5 FHETCKKIMQEEE---------------ARPRKRDGG----NFKVVCEASKILKSKGKNL

AT2G33290_SUVH2 YESLRIHLMAESM-----------------KNHVLGQGR--RRRSDMAAAYIMRDRGLWL

AT4G13460_SUVH9 YDSLRMFLMMEEA-----------------KRNGVGGRRARADGKAGKAGSMMRDCMLWM

Solyc12g096990 YDSLRIFVNLEDE-----------------NSQHLGSGRQTRARGDLKASQMMREHGLWL

Solyc09g090810 YDSLRILAMVEDD-----------------GSQHLGPYR--KPRGDLKACQILREHGLWM

AT1G17770_SUVH7 FDAVRRRLCQINH----------------------------PEDILTTASGNCTKMGVKT

AT2G24740_SUVH8 FDAVRRRLCQLNY----------------------------RKDKILTASTNCMNLGVRT

Solyc10g077070_HAP YDLFRRRMTQIEE-----------------RRGETPGS---ARRPDLKGANLLMTRGART

Solyc09g082050 FDLFRRRMTQIDE-----------------PRYGAGS----GRRPDLKASKMMMLKGMRT

AT1G73100_SUVH3 FDAVRRRLSQVEF----------------------------TKSATSKAAGTLMSNGVRT

AT5G04940_SUVH1 FDALRRRFAQLED-----------------AKEAVSGI---IKRPDLKSGSTCMGRGVRT

: . .

AT5G13960_SUVH4/KYP YP-RKIIGDLPGIDVGHRFFSRAEMCAVGFHNHWLNGIDYMSMEYEKEYSNYKL--PLAV

Solyc02g094520 N--RERIGSLPGIDVGHQFFSRAEMVVAGFHNHWLNGIDCVGQSAGKKGEYKGYSLPLAV

Solyc03g093760 NC-EWTFGHVPGVQIGDRFRFRAELVMIGLHHQFMNGINYVNIGRK----------YVAT

Solyc03g093700 NC-EWTFGHVPGVQIGDQFRFRAELVMIGLHHQFIKGINYVTIGRK----------DVAS

Solyc03g093710 NC-EWTFGHVPGVQIGDQFRFRAELVAIGLHHQFIKGINYVTIGRK----------NVAS

Solyc08g077940 NTGTQILGEVPGVEVGDAFQYRVELSLVGVHRLYQAGIDSMYIKGGL---------LVAT

Solyc06g060960 NTGQHILGEVPGVEVGDEFQYRVELAIVGVHRLYQAGIDYMKQGGM----------LIAI

AT2G22740_SUVH6 NSGVHILGEVPGVEVGDEFQYRMELNILGIHKPSQAGIDYMKYGKA----------KVAT

AT2G35160_SUVH5 YSGTQIIGTVPGVEVGDEFQYRMELNLLGIHRPSQSGIDYMKDDGGE---------LVAT

AT2G33290_SUVH2 NYDKHIVGPVTGVEVGDIFFYRMELCVLGLHGQTQAGIDCLTAERSATGE------PIAT

AT4G13460_SUVH9 NRDKRIVGSIPGVQVGDIFFFRFELCVMGLHGHPQSGIDFLTGSLSSNGE------PIAT

Solyc12g096990 NRDKRTVGPIPGVLVGDLFLYRMELCVVGLHGTPQAGIDYLPANQSSNGE------PIAT

Solyc09g090810 NRDKRIVGPIPGVLIGDVFFFRMELLVVGLHGQAQAGIDYVPASQSSNRE------PIAT

AT1G17770_SUVH7 NT-RRRIGAVPGIHVGDIFYYWGEMCLVGLHKSNYGGIDFFTAAESAVEG------HAAM

AT2G24740_SUVH8 NM-TRRIGPIPGVQVGDIFYYWCEMCLVGLHRNTAGGIDSLLAKESGVDG------PAAT

Solyc10g077070_HAP NQ-TKRIGNVPGVEVGDIFFFRMELCLVGLHAPSMAGIDYMSVRLTGDEE------PIAV

Solyc09g082050 NQ-TKRIGNVPGIEVGDIFFFRMELCVVGLHAPTMSGIDYMSLKLTKDEE------PLAV

AT1G73100_SUVH3 NM-KKRVGTVPGIEVGDIFFSRIEMCLVGLHMQTMAGIDYIISKAGSDEE------SLAT

AT5G04940_SUVH1 NT-KKRPGIVPGVEIGDVFFFRFEMCLVGLHSPSMAGIDYLVVKGETEEE------PIAT

* :.*: :* * . *: *.* **: . *

AT5G13960_SUVH4/KYP SIVMSGQYEDDLDNADTVTYTGQGGHNLTGNKR-----QIKDQLLERGNLALKHCCEYNV

Solyc02g094520 SIVVSGQYEDDQDNYEEVVYTGQGGNDLLGNKR-----QIKDQVMERGNLGLKNCMEQSV

Solyc03g093760 SIVDSGRYDNEAISSETFIYVGQGGNPKVSINA-----RVEDQKLKGGNLALKNSMDMGC

Solyc03g093700 SIVDSGRYDNEAISSETFIYVGQGGNPKVSVNA-----RMEDQKLEGGNLALKNSMELGY

Solyc03g093710 SVVDSSRYDNEAISSETFIYVGQGGNPMVSLNG-----RVEDQKLEGGNLALKNSMDLGY

Solyc08g077940 SIVASGAYDDDLGDADELIYSGQGGNV-VGKVK-----IPEDQKLVKGNLALKNSIRERN

Solyc06g060960 SIVSSGVYDDGLEDADVLIYSGQGGNV-VGKSK-----TPEDQKLERGNLALKNSISVKN

AT2G22740_SUVH6 SIVASGGYDDHLDNSDVLTYTGQGGNVMQVKKKGEELKEPEDQKLITGNLALATSIEKQT

AT2G35160_SUVH5 SIVSSGGYNDVLDNSDVLIYTGQGGNV--GKKKNNE--PPKDQQLVTGNLALKNSINKKN

AT2G33290_SUVH2 SIVVSGGYEDDEDTGDVLVYTGHGGQD--HQHK-----QCDNQRLVGGNLGMERSMHYGI

AT4G13460_SUVH9 SVIVSGGYEDDDDQGDVIMYTGQGGQD--RLGR-----QAEHQRLEGGNLAMERSMYYGI

Solyc12g096990 SIIASGGYEDDEDAGDVIIYTGQGGQD--KNSR-----QVVHQKLEGGNLALERSMYYGV

Solyc09g090810 SVIVSGGYEDDQDGGDVIIYTGHGGQD--KHSR-----QCVHQKLECGNLALERSMHYGI

AT1G17770_SUVH7 CVVTAGQYDGETEGLDTLIYSGQGGTDVYGNAR--------DQEMKGGNLALEASVSKGN

AT2G24740_SUVH8 SVVTSGKYDNETEDLETLIYSGHGG-------------KPCDQVLQRGNRALEASVRRRN

Solyc10g077070_HAP SIVSSGGYDDEGDDGEVLIYTGQGGVQ--RRDG-----QMFDQKLERGNLALEKSMHRGN

Solyc09g082050 SIVSAGGYDDDGGDGDLLIYTGQGGVQ--RKDG-----QMFDQKLEKGNLALEKSVHRAN

AT1G73100_SUVH3 SIVSSGRYEGEAQDPESLIYSGQGGNA--DKNR-----QASDQKLERGNLALENSLRKGN

AT5G04940_SUVH1 SIVSSGYYDNDEGNPDVLIYTGQGGNA--DKDK-----QSSDQKLERGNLALEKSLRRDS

.:: :. *:. : . * *:** * : ** .: .

AT5G13960_SUVH4/KYP PVRVTRGHNCKSSY---TKR-----VYTYDGLYKVEKFWAQKGVSG-FTVYKYRLKRLEG

Solyc02g094520 PVRVTRGHRCVNSY---VGK-----VYTYDGLYKVVNYWAEKGISG-FTVYKFRLKRIEG

Solyc03g093760 PVRVICGRKRVNGEKS-DIR------YIYDGLYTVTKCWEEIAPTG-KYVFKFELKRNPG

Solyc03g093700 PVRVICGRQRVNGEKS-DTR------YIYDGLYTVTKCWEERAPTG-KYIFKFELKRNLG

Solyc03g093710 PVRVICGRQRLNGEKS-DTR------YIYDGLYTVTKCWEERASTE-KYIFKFELKRNLG

Solyc08g077940 SVRVIRGSKEIRTPES-GGRPNVVTTYVYDGLYTVENYWKEKGPHG-KMVFMFKLVRIPG

Solyc06g060960 PVRVIRGSKETKNSDSVDGKGKLVTTYVYDGLYTVENYWTEQGTKG-KMVFMFKLVRVPG

AT2G22740_SUVH6 PVRVIRGKHKSTHDKS-KGG-----NYVYDGLYLVEKYWQQVGSHG-MNVFKFQLRRIPG

AT2G35160_SUVH5 PVRVIRGIKNTTLQSSVVAK-----NYVYDGLYLVEEYWEETGSHG-KLVFKFKLRRIPG

AT2G33290_SUVH2 EVRVIRGIKYENSI---SSK-----VYVYDGLYKIVDWWFAVGKSG-FGVFKFRLVRIEG

AT4G13460_SUVH9 EVRVIRGLKYENEV---SSR-----VYVYDGLFRIVDSWFDVGKSG-FGVFKYRLERIEG

Solyc12g096990 EVRVIRGFKYVGSS---SGK-----VYVYDGLYRITESWFDVGKSG-FGVYKYKLVRIEN

Solyc09g090810 EVRVIRGFKYEGSGSA-SGK-----VYVYDGLYRIVECWFDVGKSG-FGVYKYKLVRIEN

AT1G17770_SUVH7 DVRVVRGVIHPHEN---NQK-----IYIYDGMYLVSKFWTVTGKSG-FKEFRFKLVRKPN

AT2G24740_SUVH8 EVRVIRGELYN------NEK-----VYIYDGLYLVSDCWQVTGKSG-FKEYRFKLLRKPG

Solyc10g077070_HAP EVRVIRGVVDVQNGG--RGK-----IYMYDGLYRVQESWAEKSKLGNCSIFRYKLIRVPG

Solyc09g082050 DVRVIRGVKDVANP---TGK-----IYIFDGLYKIQGSWEEKIKTG-CNVFKYKLLRVPG

AT1G73100_SUVH3 GVRVVRGEEDAASK---TGK-----IYIYDGLYSISESWVEKGKSG-CNTFKYKLVRQPG

AT5G04940_SUVH1 AVRVIRGLKEASH----NAK-----IYIYDGLYEIKESWVEKGKSG-HNTFKYKLVRAPG

*** * * :**:: : * : : * * .

AT5G13960_SUVH4/KYP QPELTTDQVNF-VAG---------RIPTSTSEIEGLVCEDISGGLEFKGIPATNRVDDSP

Solyc02g094520 QPVLTTNQVHF-TRG---------CTPNSISEIRGLVCEDISGGLEDIPIPATNLVDDPP

Solyc03g093760 QPKLNREVVSRPTSLGKVDHFHVNKATKSIMESEFVVDNDVSQGKEKIPICVVNAIDDER

Solyc03g093700 QPKLARELVSRPAKLVKVNQFCVNKAKKSILQSEFVVDYDVSQGKEKIPILVVNAIDDER

Solyc03g093710 QPKLNRELVSRPAKLVKVTHSCVNKSTKSVMQSEFVVDYDVSQGKEKIPIRVVNAIDDER

Solyc08g077940 QPEL--TWKEV-QSS---------KN---SKARHGVCVPDITEGKESLPIAAVNTIDGEK

Solyc06g060960 QPEL--AWKEV-KSS---------RK---SKVRHGVCVHDITDGKETFAISAVNTIDGEK

AT2G22740_SUVH6 QPEL--SWVEV-KKS---------K----SKYREGLCKLDISEGKEQSPISAVNEIDDEK

AT2G35160_SUVH5 QPEL--PWKEV-AKS---------KK---SEFRDGLCNVDITEGKETLPICAVNNLDDEK

AT2G33290_SUVH2 QPMMGSAVMRFAQTL---------RNKPSMVRPTGYVSFDLSNKKENVPVFLYNDVDGDQ

AT4G13460_SUVH9 QAEMGSSVLKFARTL---------KTNPLSVRPRGYINFDISNGKENVPVYLFNDIDSDQ

Solyc12g096990 QPDMGSAILRFAESL---------RTRPLEVRPMGYISLDISRKKENVPVFLFNDIDNER

Solyc09g090810 QEEMGSAILRFAQNL---------RIRPLEARPTGYVTLDISRKKENVPVFLFNDIDDNH

AT1G17770_SUVH7 QPPAYAIWKTV-ENL---------RNHDLIDSRQGFILEDLSFGAELLRVPLVNEVDEDD

AT2G24740_SUVH8 QPPGYAIWKLV-ENL---------RNHELIDPRQGFILGDLSFGEEGLRVPLVNEVDEED

Solyc10g077070_HAP QPEAYTLWKSV-QQW---------REG--TATRVGVILPDLTSGAESQPVCLVNDVDDEK

Solyc09g082050 QPEAFKVWKSI-QQW---------RDG--VVSRVGVILPDLTSGAESQAVCLVNDVDDEK

AT1G73100_SUVH3 QPPAFGFWKSV-QKW---------KEG--LTTRPGLILPDLTSGAESKPVSLVNDVDEDK

AT5G04940_SUVH1 QPPAFASWTAI-QKW---------KTG--VPSRQGLILPDMTSGVESIPVSLVNEVDTDN

* *:: * : * :*

AT5G13960_SUVH4/KYP VSPTSGFTYIKSLIIEP--NVI--IPKSSTGC-NCRGSCT--DSKKCACAKLNGGNFPYV

Solyc02g094520 AAP-SGFTYSRDIVCAK--GIK--FPSAPTGC-NCHGSCL--DPRVCSCAKLNGSEFPYV

Solyc03g093760 LPS---FTYITSIRYPD--WYY---ISKPQGC-NCTSGCS--DSEQCSCASRNGGEIPF-

Solyc03g093700 PSP---FTYITSMQYPD--WYY---ISRPQGC-NCTSGCL--DSEQCSCASRNGGEIPF-

Solyc03g093710 LPP---FTYITNMQYPD--WYY---ISRPQGC-NCTSGCS--DSEQCSCASRNGGEIPF-

Solyc08g077940 PPP---FKYIKNMMYPV--GFR---PAPPRGC-DCIGRCS--DAERCSCAVKNGGEIPY-

Solyc06g060960 PPP---FNYIQKIIYPD--WFQ---PSPFKGC-DCIGRCS--DSKKCSCAVKNGGEIPY-

AT2G22740_SUVH6 PPL---FTYTVKLIYPD--WCR---PVPPKSC-CCTTRCTEAEARVCACVEKNGGEIPY-

AT2G35160_SUVH5 PPP---FIYTAKMIYPD--WCR---PIPPKSC-GCTNGCS--KSKNCACIVKNGGKIPY-

AT2G33290_SUVH2 EPR--HYEYIAKAVFPP--GIFGQGGISRTGC-ECKLSCT--D--DCLCARKNGGEFAY-

AT4G13460_SUVH9 EPL--YYEYLAQTSFPP--GLFVQQSGNASGC-DCVNGCG----SGCLCEAKNSGEIAY-

Solyc12g096990 DPA--CYDYLLKTVFPP--YVY-QHVGNGSGC-ECTDGCG--NGTNCFCAMKNGGQFAY-

Solyc09g090810 DPA--YFEYLVKPIYPP--HVS-LNVHSGNGC-QCIDGCA--D--NCFCAMRNGGQFAY-

AT1G17770_SUVH7 KTIPEDFDYIPSQCHSGMMTHEFHFDRQSLGCQNCRHQPC--MHQNCTCVQRNGDLLPY-

AT2G24740_SUVH8 KTIPDDFDYIRSQCYSG--------MTNDVNV-DSQSLVQSYIHQNCTCILKNCGQLPY-

Solyc10g077070_HAP GPA--YFTYIPSLKYSK--PFM--KSNPSVGC-QCLGGCQ-PGGTSCPCIQKNGGYLPF-

Solyc09g082050 GPA--YFTYIPSLKYSK--PFL--TPRPSLGC-QCIGGCQ-PDDTNCPCIQRNQGLLPY-

AT1G73100_SUVH3 GPA--YFTYTSSLKYSE--TFK--LTQPVIGC-SCSGSCS-PGNHNCSCIRKNDGDLPY-

AT5G04940_SUVH1 GPA--YFTYSTTVKYSE--SFK--LMQPSFGC-DCANLCK-PGNLDCHCIRKNGGDFPY-

. : * . . * * * . :.:

AT5G13960_SUVH4/KYP DLNDGRLIESRDVVFECGPHCGCGPKCVNRTSQKRLRFNLEVFRSAKKGWAVRSWEYIPA

Solyc02g094520 HKDGGRLIEPKAVVFECGPNCGCGPACVNRTSQKGLRYRLEVFRTPNKGWGVRSWDYIPS

Solyc03g093760 -NTRGSIIRAQPLVYECGPSCKCPPSCKNRVSQHGPRDHLEVFKTESRGWGLRSRDRVSS

Solyc03g093700 -NTRGSIVRAQPLVYECGPSCKCPPSCKNRVSQHGPRYHLEVFKTESRGWGLRSRDYVTR

Solyc03g093710 -NTRGSIVRAQPLVYECGPSCKCPPSCKNRVSQHGPRYHLEVFKTESRGWGLRSRDHVSS

Solyc08g077940 -NRNGAIVEVKPLVYECGPHCKCPPSCYNRVSQHGIKIPLEIFKTDTRGWGVRALTSISS

Solyc06g060960 -NRNGAIVEVKPLVYECGPHCKCPPSCYNRVSQHGIKVPLEIFKTNSRGWGVRALTSIPS

AT2G22740_SUVH6 -NFDGAIVGAKPTIYECGPLCKCPSSCYLRVTQHGIKLPLEIFKTKSRGWGVRCLKSIPI

AT2G35160_SUVH5 --YDGAIVEIKPLVYECGPHCKCPPSCNMRVSQHGIKIKLEIFKTESRGWGVRSLESIPI

AT2G33290_SUVH2 -DDNGHLLKGKHVVFECGEFCTCGPSCKSRVTQKGLRNRLEVFRSKETGWGVRTLDLIEA

AT4G13460_SUVH9 -DYNGTLIRQKPLIHECGSACQCPPSCRNRVTQKGLRNRLEVFRSLETGWGVRSLDVLHA

Solyc12g096990 -DTNGILLRGKPIIFECGPHCSCPPTCLNRVSQKGVRNRFEVFRSRETDWGVRSLDLLQA

Solyc09g090810 -DYNGILLRGKPLVFECGPHCRCPPTCRNRVTQKGLRNRFEVFRSRETGWGVRSLDLIQA

AT1G17770_SUVH7 --HNNILVCRKPLIYECGGSCPCPDHCPTRLVQTGLKLHLEVFKTRNCGWGLRSWDPIRA

AT2G24740_SUVH8 --HDNILVCRKPLIYECGGSCP------TRMVETGLKLHLEVFKTSNCGWGLRSWDPIRA

Solyc10g077070_HAP -NPLGVLMSYKTLVYECGSACSCPPNCRNRITQAGPKARVEVFKTKNRGWGLRSWDPIRG

Solyc09g082050 -NSLGVLMTYKNLIHECGSACSCPANCRNRMSQGGPKVRMEVFKTKNKGWGLRSWDPIRG

AT1G73100_SUVH3 -LNGVILVSRRPVIYECGPTCPCHASCKNRVIQTGLKSRLEVFKTRNRGWGLRSWDSLRA

AT5G04940_SUVH1 -TGNGILVSRKPMIYECSPSCPC-STCKNKVTQMGVKVRLEVFKTANRGWGLRSWDAIRA

:: . :.**. * . : . .*:*.: .*.:* :

AT5G13960_SUVH4/KYP GSPVCEYIGVVRRTADVD---TISDNEYIFEIDCQQTMQGLGGRQRRLRDVAVPMNNGVS

Solyc02g094520 GATICEYTGLLKKTDQID---PAADNNYVFDIDCLQTMKGLDGRERRLREVSLPGYWHND

Solyc03g093760 GSFICEYVGELLDEKEAE--SRIDNDEYLFDVG-NYD----EEIPKRNPMRNNNLKVESD

Solyc03g093700 GRFICEYVGELLDEKEAE--NRIGHDEYLFDIG-NYD----EEIPKRNVARNNNLKVESN

Solyc03g093710 GSFICEYVGELLDEKEAE--NRIDNDEYLFDIG-NYD----EEIPKRNVARNNNLKVDSN

Solyc08g077940 GTFICEYTGQLLEDTEAE--RRIGMDEYLFDIGQNYG----GYTANSSGQANQNELVEEG

Solyc06g060960 GTFICEYVGELLEDKEAE--QRIGSDEYLFDIGQNYS----DCSVNSSRQAEVSEVVEEG

AT2G22740_SUVH6 GSFICEYVGELLEDSEAE--RRIGNDEYLFDIGNRYD----NSLAQGMSELMLGTQAGRS

AT2G35160_SUVH5 GSFICEYAGELLEDKQAE--SLTGKDEYLFDLG---------------------------

AT2G33290_SUVH2 GAFICEYAGVVVTRLQAE-ILSMNGDVMVYPG--RFT----D-QWRNWGDLSQVYPDFVR

AT4G13460_SUVH9 GAFICEYAGVALTREQAN-ILTMNGDTLVYPA--RFS----SARWEDWGDLSQVLADFER

Solyc12g096990 GSFICEYTGVVLTQEQAQ-IFTMNGDSLIYPS--HFA----E-RWAEWGDLSRIDSNYAR

Solyc09g090810 GSFICEYTGVVLTREQAQ-IFTMNGDSLVYPS--RFP----D-RWAEWGDLSQIYPNYER

AT1G17770_SUVH7 GTFICEFAGLRKTKEEVE-----EDDDYLFDTSKIYQ----RFRWNYEPELLLEDSWEQV

AT2G24740_SUVH8 GTFICEFTGVSKTKEEVE-----EDDDYLFDTSRIYH----SFRWNYEPELLCEDACEQV

Solyc10g077070_HAP GGFVCEYAGEVIEESRVGEFGNDGDDDYIFDATRMYE----P----------LEAVRDYN

Solyc09g082050 GCFICEYAGEVRDIGYDR------DDNYIFDATRIYE----P----------LEAVHDYN

AT1G73100_SUVH3 GSFICEYAGEVKDNGNLR--GNQEEDAYVFDTSRVFN----SFKWNYEPELVDEDPSTEV

AT5G04940_SUVH1 GSFICIYVGEAKDKSKVQ--QTMANDDYTFDTTNVYN----PFKWNYEPGLADEDACEEM

* :* : * : :

AT5G13960_SUVH4/KYP QSSEDENAPEFCIDAGSTGNFARFINHSCEPNLFVQCVLS-SHQDIRLARVVLFAADNIS

Solyc02g094520 SEKMSDGGPEYCIDAVSVGNVARFINHSCQPNLFVQCVLS-THHDIGLARVVLMAADNIP

Solyc03g093760 SLGRKDEDG-FALDAVRYGNVGRFINHSCSPNLYAQNVMY-YHGDRRVPHIMFFASKSIA

Solyc03g093700 SLTRKDEDG-FTLDALRYGNVGRFINHSCSPNLYAQNVMY-YHGDKKVPHIMFFASESIA

Solyc03g093710 SSMRKDEDG-FTLDAIRYGNVGRFINHSCSPNLYAQNVMY-YHGDKKVPHIMFFASESIA

Solyc08g077940 G---------YTIDAARYGNVGRFINHSCSPNLYAQNVVY-DHKDKRVPHIMLFAADNIP

Solyc06g060960 ----------YTIDAAQYGNIGRFINHSCSPNLYAQSVLY-DHEDKKMPHIMLFAADNIP

AT2G22740_SUVH6 MAEGDESSG-FTIDAASKGNVGRFINHSCSPNLYAQNVLY-DHEDSRIPHVMFFAQDNIP

AT2G35160_SUVH5 ----DEDDP-FTINAAQKGNIGRFINHSCSPNLYAQDVLY-DHEEIRIPHIMFFALDNIP

AT2G33290_SUVH2 PNYPSLPPLDFSMDVSRMRNVACYISHSKEPNVMVQFVLH-DHNHLMFPRVMLFALENIS

AT4G13460_SUVH9 PSYPDIPPVDFAMDVSKMRNVACYISHSTDPNVIVQFVLH-DHNSLMFPRVMLFAAENIP

Solyc12g096990 PAYPSIPPLDFAMDVSRMRNLACYMSHSSSPNVLVQPVLY-DHNNVSFPHLMLFAMENIP

Solyc09g090810 PAYPSIPPLDFAMDVSRMRNVACYISHSSSPNALVQPVLY-DHNHVAFPHMMLFAMENIP

AT1G17770_SUVH7 SEFINLPTQ-VLISAKEKGNVGRFMNHSCSPNVFWQPIEY-ENRGDVYLLIGLFAMKHIP

AT2G24740_SUVH8 SEDANLPTQ-VLISAKEKGNVGRFMNHNCWPNVFWQPIEYDDNNGHIYVRIGLFAMKHIP

Solyc10g077070_HAP DESKKVPYP-LVISAKKGGNVARFMNHSCSPNVYWQLVVR-EINNETFYHVAFFAIRHIP

Solyc09g082050 DESRKVPFP-LVISSKNGGNIARFMNHSCSPNVYWQLVVR-ESNNEAYYHIAFFAIRHIP

AT1G73100_SUVH3 PEEFNLPSP-LLISAKKFGNVARFMNHSCSPNVFWQPVIR-EGNGESVIHIAFFAMRHIP

AT5G04940_SUVH1 SEESEIPLP-LIISAKNVGNVARFMNHSCSPNVFWQPVSY-ENNSQLFVHVAFFAISHIP

:. *.. ::.*. ** * : : ::* *.

AT5G13960_SUVH4/KYP PMQELTYDYGYALDSVHGPDGK---VKQLACYCGAL------------------------

Solyc02g094520 PLQELTYDYGYVLDSVMDREGK---VKQMACYCGAA------------------------

Solyc03g093760 PFEEFTYHYNYG--HVYDKNSN---MKRKNCICGSQ------------------------

Solyc03g093700 PLEELTYHYNYDVDQVSDKNGD---MKRKNCRCGSR------------------------

Solyc03g093710 PLKELTYHYNYHIDHVYDKNGD---VKRKNCRCGSR------------------------

Solyc08g077940 PLKELSYHYNYVVDQVYDSDGK---IKVKRCFCGSS------------------------

Solyc06g060960 PLAELSYHYNYSVDQVHDSKGN---IKVKKCFCGSS------------------------

AT2G22740_SUVH6 PLQELCYDYNYALDQVRDSKGN---IKQKPCFCGAA------------------------

AT2G35160_SUVH5 PLQELSYDYNYKIDQVYDSNGN---IKKKFCYCGSA------------------------

AT2G33290_SUVH2 PLAELSLDYGLADE-----------VNGKLAICN--------------------------

AT4G13460_SUVH9 PMTELSLDYGVVDD-----------WNAKLAICN--------------------------

Solyc12g096990 PLRELSIDYGMPDD-----------CTGKLAICN--------------------------

Solyc09g090810 PLKEISIDYGVADE-----------WTGNLKMMRQSCFSIPVKLKIVMTFIVVFLLQLPI

AT1G17770_SUVH7 PMTELTYDYGVSCVERSEEDEVLLYKGKKTCLCGSV------------------------

AT2G24740_SUVH8 PMTELTYDYGISCVEKTGEDEVIY-KGKKICLCGSV------------------------

Solyc10g077070_HAP PMQELTFDYGMVPPDKAD-------RRRKKCLCGSL------------------------

Solyc09g082050 PLQELTFDYGMDKAD----------HRRKKCLCGSF------------------------

AT1G73100_SUVH3 PMAELTYDYGISPTSEARDESLL--HGQRTCLCGSE------------------------

AT5G04940_SUVH1 PMTELTYDYGVSRPSGTQNGNPL--YGKRKCFCGSA------------------------

*: *: *.

AT5G13960_SUVH4/KYP -----------------NCRKRLY------------------------------------

Solyc02g094520 -----------------DCRKRLF------------------------------------

Solyc03g093760 -----------------KCEGRMY------------------------------------

Solyc03g093700 -----------------KCEGRMY------------------------------------

Solyc03g093710 -----------------KCEGRMY------------------------------------

Solyc08g077940 -----------------DCSGRMY------------------------------------

Solyc06g060960 -----------------ECSGKNFHCNLFVLLTLMFAFDRINMYLFVSISSFPIAKKVGY

AT2G22740_SUVH6 -----------------VCRRRLY------------------------------------

AT2G35160_SUVH5 -----------------ECSGRLY------------------------------------

AT2G33290_SUVH2 ------------------------------------------------------------

AT4G13460_SUVH9 ------------------------------------------------------------

Solyc12g096990 ------------------------------------------------------------

Solyc09g090810 TFVLNLFVDYLRPISLKYCASLKFIWLWRLLDL---------------------------

AT1G17770_SUVH7 -----------------KCRGSFT------------------------------------

AT2G24740_SUVH8 -----------------KCRGSFG------------------------------------

Solyc10g077070_HAP -----------------NCRGYFY------------------------------------

Solyc09g082050 -----------------KCRGYFY------------------------------------

AT1G73100_SUVH3 -----------------QCRGSFG------------------------------------

AT5G04940_SUVH1 -----------------YCRGSFG------------------------------------

AT5G13960_SUVH4/KYP --------------------

Solyc02g094520 --------------------

Solyc03g093760 --------------------

Solyc03g093700 --------------------

Solyc03g093710 --------------------

Solyc08g077940 --------------------

Solyc06g060960 IVGKVHTDFAGKEKFAQGGS

AT2G22740_SUVH6 --------------------

AT2G35160_SUVH5 --------------------

AT2G33290_SUVH2 --------------------

AT4G13460_SUVH9 --------------------

Solyc12g096990 --------------------

Solyc09g090810 --------------------

AT1G17770_SUVH7 --------------------

AT2G24740_SUVH8 --------------------

Solyc10g077070_HAP --------------------

Solyc09g082050 --------------------

AT1G73100_SUVH3 --------------------

AT5G04940_SUVH1 --------------------

**Fig. S14.** Multiple alignment of HAP and other homologous Arabidopsis and tomato SET domain containing proteins. The 8 amino acid region located inside the SET domain and shared exclusively by HAP and its closely related proteins is shown in red. **Supplementary Table S1.** Summary of down-regulated genes in *hap* mutants detected in RNAseq transcriptomic analyses. RPKM values are listed for each of three replicates of wild type (WT) and *hap* mutant (MT) plants. The calculated *p*-values (*p*-val) and False Discovery Rate (FDR) is also displayed.

| Gene | WT01 | WT02 | WT03 | MT01 | MT02 | MT03 | *p*-val | FDR | Description |
| --- | --- | --- | --- | --- | --- | --- | --- | --- | --- |
| *Solyc01g006560* | 12.04 | 15.38 | 9.52 | 0.76 | 1.69 | 1.08 | 4.91E-19 | 4.25E-15 | Lipoxygenase |
| *Solyc01g008420* | 4.00 | 2.59 | 3.42 | 0.12 | 0.08 | 0.12 | 8.47E-16 | 3.26E-12 | Mate efflux family protein |
| *Solyc01g014320* | 5.88 | 4.68 | 4.71 | 0.80 | 0.45 | 1.30 | 3.14E-05 | 5.41E-03 | SAM dependent carboxyl methyltransferase |
| *Solyc01g068460* | 18.48 | 27.31 | 25.89 | 4.47 | 6.09 | 1.96 | 3.01E-11 | 4.97E-08 | Pathogen-induced calmodulin-binding protein |
| *Solyc01g079950* | 10.38 | 16.17 | 12.33 | 3.50 | 3.18 | 2.54 | 7.41E-07 | 2.89E-04 | Xylanase inhibitor |
| *Solyc01g080220* | 4.67 | 4.45 | 5.55 | 0.35 | 0.67 | 0.63 | 4.83E-09 | 5.08E-06 | Dienelactone hydrolase |
| *Solyc01g080790* | 5.07 | 3.79 | 2.95 | 0 | 0.13 | 0 | 7.63E-14 | 2.04E-10 | Plant Basic Secretory Protein |
| *Solyc01g090300* | 6.09 | 9.22 | 4.48 | 0.78 | 1.40 | 0.98 | 1.81E-06 | 5.49E-04 | Ethylene responsive transcription factor 1b |
| *Solyc01g104780* | 19.86 | 22.58 | 20.83 | 3.76 | 7.15 | 4.67 | 2.27E-07 | 1.18E-04 | Nodulin-related integral membrane protein DUF125 |
| *Solyc02g005110* | 70.70 | 80.96 | 95.17 | 17.64 | 20.39 | 23.73 | 1.89E-06 | 5.65E-04 | Unknown Protein |
| *Solyc02g071380* | 4.93 | 4.38 | 6.39 | 0.36 | 0.26 | 0.35 | 1.75E-13 | 4.05E-10 | 1-aminocyclopropane-1-carboxylate oxidase 3 |
| *Solyc02g075620* | 2.54 | 2.50 | 3.02 | 0 | 0.40 | 0.36 | 5.31E-07 | 2.33E-04 | Pectinesterase |
| *Solyc02g079490* | 12.25 | 7.47 | 10.79 | 1.74 | 2.78 | 2.62 | 5.45E-07 | 2.35E-04 | Hydroxycinnamoyl-CoA shikimate/quinate hydroxycinnamoyl transferase |
| *Solyc02g082450* | 3.29 | 2.74 | 6.28 | 0.31 | 0.55 | 0.77 | 2.21E-06 | 6.47E-04 | Auxin efflux carrier family protein |
| *Solyc03g097870* | 49.84 | 23.40 | 38.85 | 3.91 | 8.39 | 3.84 | 2.93E-13 | 5.98E-10 | MtN3-like protein_homologous SWEET11_sugar transsporter |
| *Solyc03g098300* | 22.99 | 42.11 | 20.29 | 2.96 | 4.13 | 3.57 | 2.63E-13 | 5.71E-10 | Ornithine decarboxylase |
| *Solyc04g007400* | 43.30 | 43.20 | 38.29 | 9.12 | 14.90 | 11.24 | 1.19E-08 | 1.12E-05 | Tropinone reductase II |
| *Solyc04g007790* | 307.90 | 270.20 | 305.10 | 33.60 | 14.70 | 13.46 | 9.29E-19 | 6.45E-15 | Major latex-like protein |
| *Solyc04g052980* | 9.38 | 12.04 | 8.90 | 1.00 | 2.84 | 1.57 | 2.74E-06 | 7.72E-04 | Auxin-responsive protein |
| *Solyc04g057940* | 5.06 | 4.79 | 5.27 | 0.95 | 1.69 | 0.59 | 1.64E-08 | 1.39E-05 | U-box domain-containing protein |
| *Solyc04g057980* | 50.37 | 42.33 | 51.36 | 7.62 | 19.39 | 4.38 | 9.99E-08 | 5.92E-05 | NAD(P)H-quinone oxidoreductase subunit M |
| *Solyc04g078030* | 2.93 | 5.11 | 3.98 | 0.37 | 0.89 | 0.45 | 4.68E-06 | 1.24E-03 | Unknown Protein |
| *Solyc05g015780* | 24.65 | 26.28 | 19.08 | 5.01 | 6.67 | 3.90 | 1.19E-04 | 1.31E-02 | Unknown Protein |
| *Solyc05g055290* | 4.54 | 3.53 | 3.02 | 0 | 0.09 | 0 | 1.89E-17 | 9.36E-14 | Hydrolase alpha/beta fold family protein |
| *Solyc06g009190* | 35.09 | 35.24 | 39.09 | 7.55 | 15.58 | 4.74 | 2.97E-08 | 2.29E-05 | Pectinesterase |
| *Solyc06g010240* | 3.09 | 6.39 | 5.83 | 0 | 0.80 | 0.82 | 7.49E-04 | 4.24E-02 | Unknown Protein |
| *Solyc06g049020* | 19.58 | 29.76 | 20.42 | 6.32 | 6.82 | 7.53 | 6.71E-05 | 9.16E-03 | Unknown Protein |
| *Solyc06g051560* | 7.83 | 15.43 | 9.52 | 0.96 | 3.28 | 2.24 | 1.27E-04 | 1.37E-02 | Flavoprotein wrbA |
| *Solyc06g052010* | 5.36 | 4.18 | 6.45 | 0.79 | 1.51 | 0.23 | 4.42E-06 | 1.18E-03 | IQ calmodulin-binding region |
| *Solyc06g069070* | 134.20 | 128.80 | 108.50 | 10.54 | 17.83 | 4.55 | 4.25E-17 | 1.84E-13 | Lipid transfer protein |
| *Solyc06g071830* | 13.58 | 11.49 | 13.32 | 1.60 | 4.01 | 2.83 | 1.67E-07 | 9.21E-05 | BTB/POZ domain-containing protein |
| *Solyc06g082300* | 7.46 | 5.46 | 8.39 | 0.80 | 1.76 | 1.77 | 1.49E-06 | 4.69E-04 | UDP-glucosyltransferase |
| *Solyc07g005370* | 143.90 | 86.15 | 121.40 | 13.57 | 21.25 | 21.200 | 4.16E-15 | 1.31E-11 | Norcoclaurine synthase |
| *Solyc07g006680* | 20.77 | 14.41 | 22.43 | 4.75 | 4.71 | 3.10 | 8.72E-09 | 8.39E-06 | Hydroxycinnamoyl CoA quinate transferase |
| *Solyc07g007260* | 138.60 | 134.30 | 136.40 | 16.42 | 66.06 | 18.95 | 5.93E-07 | 2.51E-04 | Metallocarboxypeptidase inhibitor |
| *Solyc07g021530* | 5.15 | 7.16 | 12.41 | 0.31 | 0.58 | 0.90 | 2.71E-06 | 7.69E-04 | Unknown Protein |
| *Solyc07g040960* | 7.45 | 12.36 | 19.34 | 2.11 | 1.98 | 1.95 | 1.38E-08 | 1.22E-05 | Unknown protein |
| *Solyc07g042630* | 50.07 | 38.24 | 43.04 | 3.06 | 6.42 | 2.31 | 6.46E-24 | 1.36E-19 | Beta-Amyrin Synthase |
| *Solyc07g048060* | 11.17 | 20.10 | 10.70 | 0.32 | 2.46 | 0.10 | 1.75E-07 | 9.37E-05 | Auxin-induced protein-like |
| *Solyc07g053550* | 12.89 | 46.41 | 12.20 | 4.85 | 4.38 | 1.69 | 4.61E-05 | 6.98E-03 | Glutaredoxin |
| *Solyc07g063770* | 3.83 | 4.91 | 2.79 | 0.56 | 0.81 | 0.62 | 9.32E-08 | 5.67E-05 | Serine/threonine kinase receptor |
| *Solyc08g007040* | 289.30 | 262.90 | 275.60 | 52.33 | 50.19 | 103.5 | 8.23E-07 | 3.08E-04 | Glycine cleavage system H protein 1 |
| *Solyc08g007680* | 6.40 | 6.58 | 7.26 | 1.84 | 2.60 | 1.38 | 7.98E-06 | 1.81E-03 | Subtilisin-like protease |
| *Solyc08g007690* | 4.23 | 3.68 | 3.42 | 0.38 | 0.86 | 0.46 | 7.20E-09 | 7.14E-06 | Subtilisin-like protease |
| *Solyc08g008500* | 8.83 | 15.00 | 10.40 | 0.29 | 3.39 | 0.49 | 8.31E-06 | 1.87E-03 | Dof zinc finger protein |
| *Solyc08g028690* | 18.80 | 19.86 | 25.94 | 5.16 | 8.60 | 6.92 | 1.96E-05 | 3.74E-03 | Tasselseed2-like short-chain dehydrogenase/reductase |
| *Solyc08g078330* | 14.22 | 16.67 | 17.85 | 0.87 | 2.90 | 2.27 | 6.16E-11 | 9.72E-08 | Oxidoreductase 2OG-Fe(II) oxygenase family |
| *Solyc09g010570* | 11.34 | 9.92 | 10.85 | 2.60 | 3.49 | 1.04 | 2.28E-05 | 4.14E-03 | Unknown Protein |
| *Solyc09g065620* | 2.54 | 3.11 | 3.78 | 0.14 | 0.09 | 0.41 | 1.26E-08 | 1.15E-05 | Chlorophyllase 1 |
| *Solyc09g075710* | 3.43 | 4.15 | 3.07 | 0.23 | 0.91 | 0.16 | 9.74E-07 | 3.42E-04 | Gibberellin receptor GID1L2 |
| *Solyc09g082700* | 68.64 | 45.79 | 50.51 | 1.80 | 4.82 | 4.76 | 3.25E-20 | 3.75E-16 | Early light-induced protein |
| *Solyc09g092490* | 20.12 | 14.68 | 21.25 | 3.63 | 5.42 | 5.78 | 6.15E-06 | 1.50E-03 | UDP-glucosyltransferase family 1 protein |
| *Solyc10g006860* | 21.98 | 20.38 | 21.82 | 3.21 | 4.56 | 6.14 | 6.68E-08 | 4.29E-05 | Glucose/ribitol dehydrogenase |
| *Solyc10g006900* | 61.77 | 58.04 | 65.98 | 6.42 | 22.55 | 15.51 | 4.09E-08 | 3.02E-05 | Protochlorophyllide reductase |
| *Solyc10g007110* | 31.68 | 30.36 | 32.26 | 10.85 | 14.07 | 11.08 | 8.84E-06 | 1.94E-03 | Tyrosine aminotransferase |
| *Solyc10g007750* | 3.14 | 2.72 | 2.46 | 0.20 | 0.38 | 0.13 | 4.42E-08 | 3.19E-05 | Plant Basic Secretory Protein |
| *Solyc10g008160* | 3.71 | 4.46 | 5.60 | 0.75 | 1.24 | 0.45 | 1.48E-05 | 3.00E-03 | Transcription factor |
| *Solyc10g018140* | 9.09 | 7.89 | 12.05 | 0.44 | 0.56 | 0.07 | 4.15E-15 | 1.31E-11 | Dihydroflavonol 4-reductase |
| *Solyc10g051020* | 8.41 | 5.94 | 4.88 | 0.39 | 0.65 | 0.35 | 9.25E-14 | 2.29E-10 | Cytochrome P450 |
| *Solyc10g077070* | 51.78 | 45.76 | 49.67 | 10.69 | 10.71 | 10.73 | 5.35E-13 | 1.03E-09 | HAIRPLUS |
| *Solyc10g078260* | 14.47 | 10.53 | 12.45 | 0.90 | 4.56 | 0.42 | 1.37E-05 | 2.82E-03 | Peptidyl-prolyl cis-trans isomerase |
| *Solyc10g080340* | 6.45 | 6.38 | 7.50 | 0.69 | 1.98 | 0.60 | 6.02E-08 | 4.06E-05 | Multi antimicrobial extrusion protein MatE |
| *Solyc10g085870* | 25.02 | 14.69 | 15.51 | 4.11 | 5.83 | 3.03 | 5.46E-08 | 3.86E-05 | UDP-glucosyltransferase family 1 protein |
| *Solyc10g085880* | 3.43 | 1.69 | 1.43 | 0.11 | 0 | 0.16 | 6.49E-08 | 4.25E-05 | UDP-glucosyltransferase family 1 protein |
| *Solyc10g086720* | 4.21 | 3.91 | 4.93 | 0.24 | 1.33 | 1.30 | 4.21E-04 | 2.94E-02 | Fructose-1 6-bisphosphatase class 1 |
| *Solyc11g005340* | 28.24 | 22.62 | 24.56 | 4.91 | 8.03 | 5.40 | 6.86E-08 | 4.33E-05 | PAP fibrillin family protein |
| *Solyc11g007730* | 33.62 | 23.62 | 28.98 | 4.80 | 10.92 | 4.82 | 2.09E-08 | 1.69E-05 | MTA/SAH nucleosidase / phosphatase |
| *Solyc11g011740* | 3.84 | 2.08 | 4.35 | 0.21 | 0.20 | 0.31 | 2.52E-07 | 1.28E-04 | Ethylene-responsive transcription factor 2 |
| *Solyc11g064840* | 5.97 | 6.59 | 5.10 | 1.14 | 1.40 | 0.51 | 6.47E-07 | 2.61E-04 | C2 calcium-dependent membrane targeting |
| *Solyc11g068380* | 6.74 | 9.83 | 7.17 | 0.78 | 1.90 | 0.86 | 7.15E-07 | 2.82E-04 | Unknown Protein |
| *Solyc11g071290* | 7.62 | 13.30 | 5.79 | 0.20 | 1.59 | 0.19 | 5.61E-08 | 3.89E-05 | Alcohol dehydrogenase |
| *Solyc12g009800* | 148.20 | 149.10 | 173.10 | 38.68 | 47.35 | 34.75 | 1.66E-11 | 2.88E-  08 | Purple acid phosphatase 3 |
| *Solyc12g010950* | 56.87 | 51.57 | 47.90 | 3.37 | 6.00 | 5.50 | 7.83E-24 | 1.36E-19 | Alcohol dehydrogenase zinc-containing |
| *Solyc12g011040* | 9.47 | 12.00 | 10.37 | 1.56 | 1.66 | 0.71 | 6.00E-14 | 1.73E-10 | Lipoxygenase |
| *Solyc12g042100* | 15.90 | 17.71 | 16.34 | 3.35 | 5.39 | 1.00 | 4.40E-05 | 6.84E-03 | Unknown Protein |
| *Solyc12g056620* | 16.07 | 11.15 | 15.12 | 2.95 | 4.38 | 4.59 | 2.12E-04 | 1.88E-02 | One-helix protein |
| *Solyc12g088700* | 14.27 | 10.83 | 13.90 | 2.48 | 3.87 | 4.08 | 7.69E-06 | 1.76E-03 | UDP-glucosyltransferase family 1 protein |
| *Solyc12g089380* | 5.79 | 10.89 | 9.45 | 0.29 | 1.09 | 0.84 | 8.10E-10 | 1.04E-06 | Expansin 45, endoglucanase-like |
| *Solyc12g094460* | 18.30 | 19.15 | 18.25 | 7.59 | 4.34 | 5.52 | 5.26E-05 | 7.80E-03 | Laccase-2 |
| *Solyc12g098600* | 20.07 | 16.21 | 18.90 | 3.76 | 7.11 | 3.34 | 7.08E-08 | 4.38E-05 | UDP-glucosyltransferase family 1 protein |
| *Solyc12g098900* | 25.25 | 45.78 | 41.32 | 1.42 | 10.91 | 1.10 | 1.16E-06 | 3.78E-04 | Late embryogenesis abundant protein D-29 |
| *Solyc12g099430* | 22.10 | 21.81 | 24.69 | 3.15 | 2.40 | 5.00 | 3.74E-09 | 4.05E-06 | Unknown Protein |

**Supplementary Table S2.** Summary of up-regulated genes in *hap* mutants detected in RNAseq transcriptomic analyses. RPKM values are listed for each of three replicates of wild type (WT) and *hap* mutant (MT) plants. The calculated *p*-values (*p*-val) and False Discovery Rate (FDR) is also displayed.

| Genes | WT01 | WT02 | WT03 | MT01 | MT02 | MT03 | *p*-val | FDR | Description |
| --- | --- | --- | --- | --- | --- | --- | --- | --- | --- |
|  |  |  |  |  |  |  |  |  |  |
| *Solyc02g077880* | 138.70 | 158.96 | 207.30 | 782.30 | 443.88 | 765.93 | 9.92E-11 | 1.49E-07 | Auxin-repressed protein |
| *Solyc02g085910* | 0.37 | 0.59 | 0.47 | 35.48 | 2.22 | 4.39 | 3.25E-08 | 2.45E-05 | Lateral organ boundaries (LOB), LOB domain protein 42 |
| *Solyc03g020060* | 0 | 0 | 0.27 | 1.85 | 24.50 | 4.10 | 1.23E-10 | 1.77E-07 | Proteinase inhibitor I20, Pin2 |
| *Solyc03g020070* | 0.14 | 0.08 | 0.15 | 1.58 | 28.87 | 6.78 | 1.28E-11 | 2.34E-08 | Proteinase inhibitor I20, Pin2 |
| *Solyc03g020080* | 0 | 0 | 0.08 | 5.53 | 42.27 | 7.31 | 1.16E-18 | 6.69E-15 | Proteinase inhibitor I20, Pin2 |
| *Solyc03g082550* | 0.57 | 1.15 | 0.85 | 8.56 | 3.32 | 3.34 | 1.80E-06 | 5.49E-04 | Homeobox leucine zipper protein |
| *Solyc08g065940* | 58.54 | 35.83 | 45.96 | 217.31 | 131.50 | 157.06 | 1.88E-10 | 2.60E-07 | Zinc finger CCCH domain-containing protein 20 |
| *Solyc12g096570* | 10.92 | 20.34 | 14.02 | 143.37 | 50.40 | 54.20 | 1.63E-08 | 1.39E-05 | ARGOS |

**Supplementary Table S3.** Two-ways ANOVA analysis of genotype (WT and *hap*) and tissue source (vegetative stem, inflorescence stem, leaf adaxial and leaf abaxial side) influence on the density of the different trichome types and stomata*. P* < 0.01 values were considered as statistically significant.

| **Cell type** | **Genotype** | **Tissue** | **Genotype:Tissue** |
| --- | --- | --- | --- |
| Type I | 1.72E-12 | 2.06E-22 | 9.81E-15 |
| Type III | 9.47E-05 | 1.17E-11 | 1.56 E-03 |
| Type VI | 0.24 | 6.43E-12 | 0.19 |
| Type VII | 1.11E-03 | 2.47E-17 | 0.02 |
| Type V | 0.05 | 2.39E-09 | 0.15 |
| Stomata | 0.29 | 1.28E-15 | 0.33 |

**Supplementary Table S4.** Statistical differences accounted for type-I trichome density assessed by means of a Dunnett’s test comparison performed between transgenic lines and WT plants*. P* < 0.01 values were considered as statistically significant.

| **Genotype** | ***p-*value** | **Mean difference** |
| --- | --- | --- |
| *hap* | 2.90 E-06 | 4.8 |
| RNAi | 2.70E-10 | 6.8 |
| CRISPR | 2.00E-16 | 6.8 |
| OE::*HAP* WT | 1.00 | 0.0 |
| OE::*HAP* *hap* | 0.08 | -1.8 |

**Supplementary Table S5.** Primers used for expression analysis and functional characterization of *HAP.*

| **Name** | **Primer** |
| --- | --- |
| RNAi::*HAP* | F: tctagactcgagGCACTAGTCTGATGCATAAAAAGAA |
|  | R: atcgatggtaccAAGTACTTAGGATTGTCATTGACTGG |
| OE::*HAP* | F: ggtaccGGGAGAAGTGCTGACCTTTG |
|  | R: gtcgacATGTCCAAAACCTTGCTGCT |
| sgRNA-*HAP* | F: ATTGAGCATGCATCGTGGCAATG |
|  | R: AAACCATTGCCACGATGCATGCT |
| Sequencing sgRNA-*HAP* | F: TTGTGCCTGGTTGGTTTACA |
|  | R: CAGGTTGACTCTCTGCACCA |
| qRT-PCR *HAP* | F: TTATACTGGCCAGGGTGGAG |
|  | R: CAATTGCCCAATTTGCTTTT |
| HAPpro | F: gtcgacATGGAGCAAGGCTTT |
|  | R: gcggccgcCTAGTAAAAATAC |
